# Antigen experience history directs distinct functional states of CD8+ CAR T cells during the anti-leukemia response

**DOI:** 10.21203/rs.3.rs-3712137/v1

**Published:** 2023-12-21

**Authors:** Kole R. DeGolier, Etienne Danis, Marc D’Antonio, Jennifer Cimons, Michael Yarnell, Ross M. Kedl, M. Eric Kohler, James P. Scott-Browne, Terry J. Fry

**Affiliations:** 1 Department of Immunology, University of Colorado Anschutz Medical Campus; Aurora, CO, USA; 2 Department of Pediatrics, University of Colorado Anschutz Medical Campus; Aurora, CO, USA; 3 Biostatistics and Bioinformatics Shared Resource, University of Colorado Cancer Center, University of Colorado Anschutz Medical Campus; Aurora, CO, USA; 4 Center for Cancer and Blood Disorders, Children’s Hospital Colorado; Aurora, CO, USA; 5 Department of Immunology and Genomic Medicine, National Jewish Health, Denver, CO, USA

## Abstract

Chimeric antigen receptor T cells are an effective therapy for B-lineage malignancies. However, many patients relapse and this therapeutic has yet to show strong efficacy in other hematologic or solid tumors. One opportunity for improvement lies in the ability to generate T cells with desirable functional characteristics. Here, we dissect the biology of CD8+ CAR T cells (CAR8) by controlling whether the T cell has encountered cognate TCR antigen prior to CAR generation. We find that prior antigen experience influences multiple aspects of *in vitro* and *in vivo* CAR8 functionality, resulting in superior effector function and leukemia clearance in the setting of limiting target antigen density compared to antigen-inexperienced T cells. However, this comes at the expense of inferior proliferative capacity, susceptibility to phenotypic exhaustion and dysfunction, and inability to clear wildtype leukemia in the setting of limiting CAR+ cell dose. Epigenomic and transcriptomic comparisons of these cell populations identified overexpression of the Runx2 transcription factor as a novel strategy to enhance CAR8 function, with a differential impact depending on prior cell state. Collectively, our data demonstrate that prior antigen experience determines functional attributes of a CAR T cell, as well as amenability to functional enhancement by transcription factor modulation.

Adoptive transfer of T cells expressing chimeric antigen receptors (CARs) has been highly successful for treating relapsed and treatment-refractory B-lineage hematologic malignancies. However, many patients do not achieve complete remission, or relapse. Poor response or lack of remission durability results from cancer cell resistance or suboptimal CAR T cell function^1^. Thus, further studies into the immunobiology of these engineered cells are warranted to enhance remissions and expand therapeutic potential to other hematologic and solid tumors. CAR T cells are commonly generated from a heterogeneous population of peripheral blood T cells that varies between patients, likely impacting the quality of a CAR T cell product^2^. Although it has been difficult to track cell fate through the manufacturing process and into patients, previous reports have shown differential function of CAR T cell products generated from memory versus naïve T cells sorted by surface marker phenotypes, which are not always an accurate representation of cellular differentiation state^2,3,4^. Emerging studies have demonstrated that phenotypic, transcriptomic and epigenomic attributes of the CAR product can influence patient outcomes^5^. During acute infections, naïve CD8+ T cells become activated through the T cell antigen receptor (TCR) by antigen presenting cells displaying cognate antigen and co-stimulatory ligands, and subsequently enter a highly regulated differentiation trajectory. A phase of rapid expansion and differentiation into effector cells is followed by contraction and formation of long-lived memory cells that rapidly respond to future exposures. However, if the pathogen is not cleared, antigen-specific T cell populations will receive recurring antigen stimulation. In this setting, rather than forming functional memory, T cells differentiate down a trajectory characterized by progressive dysfunction, preventing immune-mediated pathology, but simultaneously failing to clear the challenge. A growing body of work demonstrates that these differentiation trajectories (and resulting functional characteristics imbued on T cells) are controlled epigenetically in traditional T cell responses to viral infections and tumors. These programs are defined by progressive changes to the epigenome, associated with DNA methylation and histone modifications which are driven by a variety of transcription factors (TFs) and modulated by antigen receptor signaling^6^. These molecular modifications alter chromatin accessibility and transcriptional profiles which characterize cellular differentiation state and functional capacity. Epigenetic modulation of T cells via stimulation through the physiologic TCR has a well-established role in impacting the differentiation program and functional capacity of a pool of antigen-experienced T cells^7^. Emerging data also highlight the importance of epigenetic remodeling in CAR T cell responses to tumors^5^.

Here, we carefully examine and compare the biology of CAR-transduced CD8+ T cells that differ as to whether cognate antigen has been encountered through the TCR prior to transduction with a CAR. We hypothesize that 1) T cells exhibit functional characteristics after CAR transduction that are dictated by prior antigen experience via the TCR 2)the functional characteristics of CAR8 derived from naïve or memory cells are the result of epigenetic attributes maintained through CAR transduction and reinfusion, and that 3) TF modulation as a modality to enhance CAR8 function may be dependent on the epigenetic and transcriptomic contexts determined by prior antigen experience status. Prior work has shown dose-dependent effects in the anti-tumor responses of adoptively-transferred T cells^2^ and CAR T cells have been shown to elicit poor responses to tumors with low antigen density^1, 8, 9, 10^. Using limiting target antigen density or limiting T cell dose as stressors, we show that prior T cell antigen experience influences *in vitro* and *in vivo* functional characteristics of T cells stimulated through a CAR. Comparison of the epigenetic and transcriptomic states of CAR8 stratified by prior antigen-experience status revealed differential chromatin accessibility and transcriptional programming. We pinpoint divergent RUNX2 activity within the two populations as a potential driver of differential function and show that ectopic expression of RUNX2 enhances the anti-leukemia response and mediates exhaustion resistance in CAR T cells in a manner dependent on prior T cell antigen experience status.

## RESULTS

### T cell antigen experience prior to transduction with a CAR directs *in vitro* proliferative and effector capacities of CD8+ CAR T cells.

**Figure 1:**
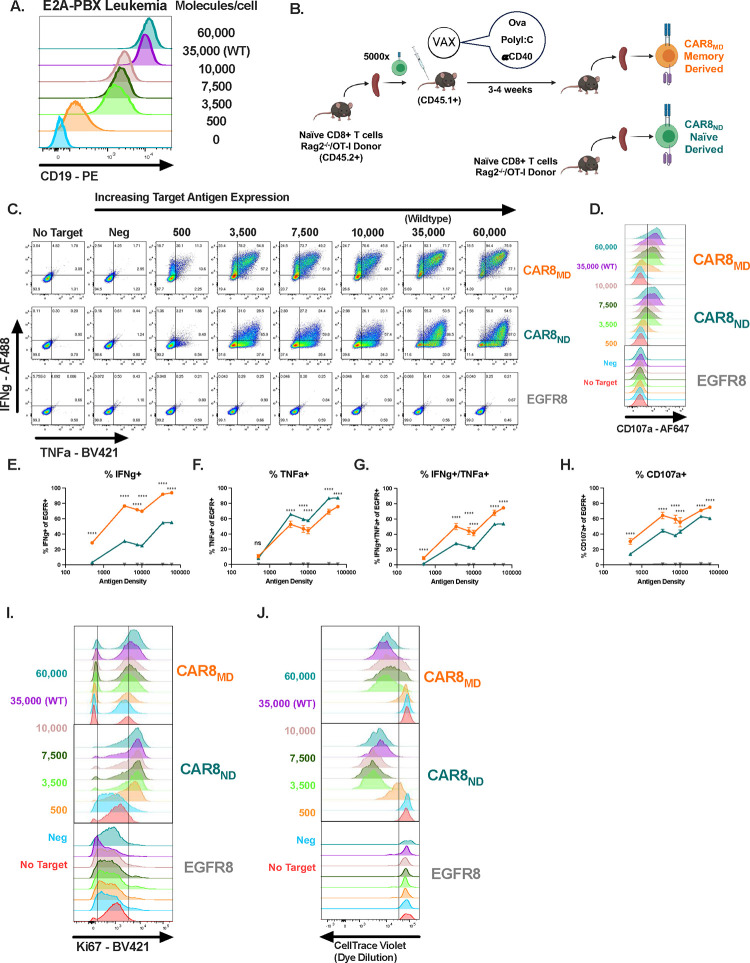
Antigen experience history directs multiple aspects of *in vitro* functional capacity of murine CD8+ CAR T cells. **1A:** E2A-PBX murine leukemia was engineered to knockout CD19, followed by reintroduction of CD19 at different levels to generate a range of antigen density clones. **1B:** Schematic: Vaccine model for generating memory CD8+ OT-I T cells. 5e3 OT-I T cells were transferred into congenically distinct hosts which were concurrently vaccinated with antigen and adjuvants. 3–5 weeks later, CAR T cells were manufactured from memory OT-I’s (CAR8_MD,_ memory-derived) or naïve OT-I’s (CAR8_ND_, naïve-derived) **1C:** Intracellular cytokine staining of IFNg and TNFa after 6 hour co-culture assay. **1D:** Degranulation as measured by CD107a expression after 4 hour co-culture assay. **1E-G:** Quantification of cytokine data, % positive cells for indicated cytokine. **1H:** Quantification of CD107a data, % positive cells. **1I:** Cell-cycle entry as measured by Ki-67 staining after 18 hour co-culture assay. **1J:** Proliferation as measured by dilution of CellTrace Violet dye after 72 hour co-culture assay. All *in vitro* assays were performed with n=3 technical replicates, and are representative of 2 independent experiments. Data represent mean +/− SD. * p<0.05, ** p<0.01, *** p<0.001, **** p<0.0001.

Memory T cells demonstrate superior antigen sensitivity compared to naïve T cells in some contexts^11,12^. Thus, we hypothesized that CAR T cells derived from a memory T cell population would exhibit enhanced responsiveness to low antigen density leukemias compared to naïve-derived CAR T cells. T cells expressing a CAR containing an anti-mouse CD19 scFv incorporating a FLAG sequence and a CD28 costimulatory domain fused to mouse CD3zeta, followed by a 2A sequence and a truncated EGFR ^13^ (Figure S1A) were used to target a murine leukemia driven by the E2A-PBX1 fusion protein (E2A-PBX)^14, 15, 16^. FLAG-specific antibody detection of the CAR correlated strongly with EGFR expression, allowing for use of EGFR as a marker for long term tracking of CAR+ cells *in vivo* (Figure S1B). We expanded this model by generating a set of clones of E2A-PBX which express differing CD19 densities (Figure 1A, S1C). Memory OT-I T cells generated using a well-characterized ovalbumin vaccination model ^17, 18, 19^ (Figure 1B). were used to produce memory-derived CD8+ CAR T cells (CAR8_MD_) for comparison to naïve-derived CD8+ OT-I CAR T cells (CAR8_ND_). As no difference was seen in leukemia control by memory or naïve-derived control T cells (Figure S1D), we used naïve-derived (EGFR8) in all subsequent experiments. A functional duality began to emerge upon *in vitro* testing. As predicted, a greater proportion of CAR8_MD_ cells had a polyfunctional effector profile, producing both TNFa and IFNg, or degranulating (as measured by CD107a), most pronounced in response to low target antigen (Figure 1C–H; S1E–G). Interestingly, while the proportion of IFNg+ cells was greater in CAR8_MD_, the proportion of TNFa+ cells was slightly increased in CAR8_ND_, suggesting a predisposition toward either IFNg or TNFa (Figure 1C & F). However, CAR8_ND_ outperformed CAR8_MD_ in cell cycle entry (Ki67 expression; Figure 1I, S1H) and extended proliferative capacity (Figure 1J, S1I) across antigen densities. To compare polyclonal antigen-experienced and naïve T cells more analogous to human CAR T cells, we generated pathogen-elicited polyclonal T cells by infecting WT C57BL/6 mice with the common acute viral infection model LCMV-Armstrong. Memory (CD8+/CD44+/CD49d^Hi^) and naïve (CD8+/CD44−/CD49d^Lo^/CD62L+) T cell populations were FACS-sorted from the same mice 28 days after LCMV infection and used for CAR T cell manufacturing (Figure S2A). Polyclonal pathogen-elicited T cells behaved similarly *in vitro* to memory and naïve OT-I cells: CAR8_MD_ demonstrated superior effector function (increased proportions of cells producing IFNg) and CAR8_ND_ demonstrated superior proliferative capacity (Figure S2B–E). Thus, CD8+ T cell antigen experience prior to transduction with a CAR promotes effector functions at the expense of proliferative capacity.

### Treatment of leukemia-bearing mice with a high CAR+ cell dose reveals enhanced cytotoxic profile and clearance of antigen-low leukemia by memory-derived CAR8.

**Figure 2:**
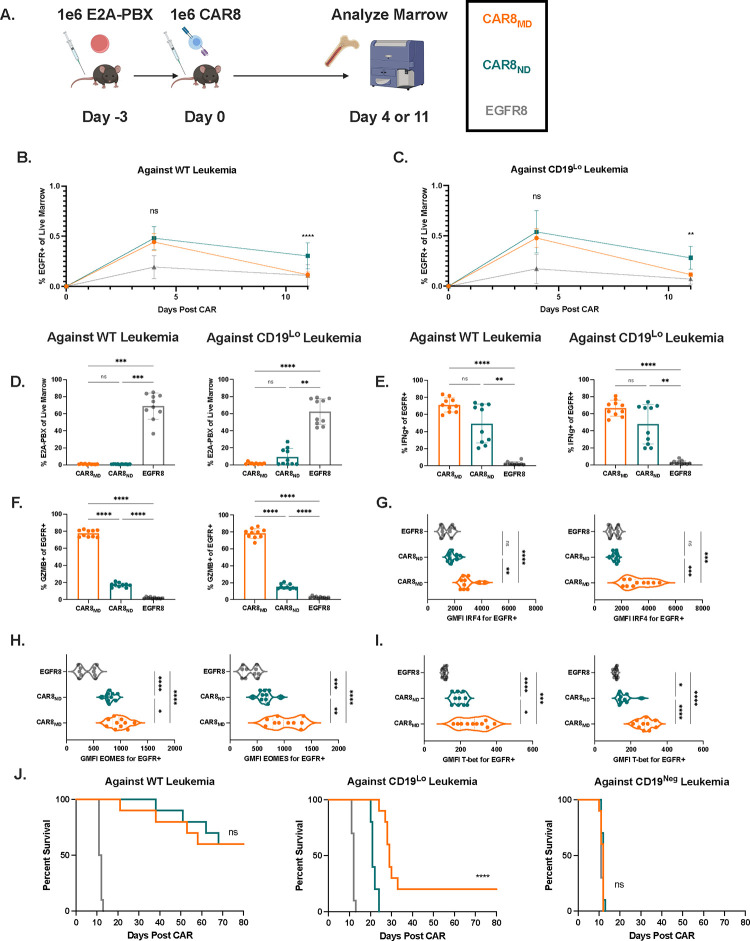
CAR8_MD_ exhibit enhanced cytotoxicity and clearance of CD19^Lo^ leukemia *in vivo* (high CAR dose). **2A:** Schematic: Timeline for *in vivo* experiments. *Rag1*^−/−^ mice were injected with 1e6 E2A-PBX1 leukemia on day −3, followed by 1e6 OT-I CD8+/EGFR+ T cells from indicated T cell condition on day 0. Bone marrow was analyzed by flow cytometry on day +4 or day +11. T cell populations were isolated memory-derived CAR T cells (CAR8_MD_), isolated naïve-derived CAR T cells (CAR8_ND_) or EGFR control T cells (EGFR8). Leukemia populations were CD19^Neg^, CD19^Lo^(10,000 antigens/cell), or WT (35,000 antigens/cell). **2B-C:** Early T cell expansion (day +4) or persistence (day +11) after infusion of transduced T cells against WT leukemia **(B)** and CD19^Lo^ leukemia **(C)**. Transduced T cell populations measured by coexpression of CD8a+/TCRbeta+/EGFR+. **2D:** Clearance of WT and CD19^Lo^ leukemia at day +11 after CAR infusion. E2A-PBX measured by coexpression of B220+/CD22+. **2E-F:** Intracellular cytokine staining of interferon gamma **(E)** or granzyme B **(F)** in CAR T cells from whole bone marrow restimulated *ex vivo* with leukemia. Data represent mean +/− SD. **2G-I:** Intranuclear transcription factor staining of IRF4 **(G)**, EOMES **(H)**, or T-bet **(I)** on CAR+ T cells from mice bearing the indicated leukemia at day +4 after CAR infusion. Violin plot data represent median with quartiles. Data are from 2 pooled, independent experiments with n=10 mice per condition. * p<0.05, ** p<0.01, *** p<0.001, **** p<0.0001. **2J:** Survival of mice after treatment with 1e6 EGFR+ CAR or control T cells. Survival statistics were performed using log-rank (Mantel-Cox) test * p<0.05, ** p<0.01, *** p<0.001, **** p<0.0001. Data is from 2 independent pooled experiments, total n=10 mice per group.

Given the opposing functional profiles of naïve and memory-derived CAR8, we next compared the ability of these two populations to mediate tumor clearance *in vivo*. Mice were engrafted with WT (35,000 antigens per cell), CD19^Lo^ (10,000 antigens per cell) or CD19^Neg^ leukemia followed 3 days later by a dose of 1e6 CAR T cells. The CD19^Lo^ clone antigen density was chosen based on differential *in vitro* responses and, although higher than antigen density reported for CAR relapses post-CD22 CAR treatment^9^, is consistent with the drop-off in CAR sensitivity against other antigens^8, 10^. *Rag1*-deficient hosts enabled CAR T cell expansion without irradiation and limited CAR T cell antigen exposure to CD19 densities expressed on leukemia rather than endogenous B cells. While we did not observe differences in proportions of CAR T cells in the marrow at peak expansion on day 4 (Figure 2A), post-contraction (day 11) CAR8_ND_ had increased proportions and total counts of CAR T cells in mice bearing WT and CD19^Lo^ leukemia (Figure 2B–C, Figure S3A–B). Both CAR groups mediated robust clearance of WT leukemia by day 11. Although there was no significant difference in clearance of CD19^Lo^ leukemia, 4/10 mice treated by CAR8_ND_ had detectable leukemia at >15% of live bone marrow cells while all 10 mice treated with CAR8_MD_ had minimal leukemic burdens (<5%) (Figure 2D). We next tested whether the enhanced clearance of CD19^Lo^ leukemia was associated with maintenance of the superior cytotoxic capacity of CAR8_MD_ observed *in vitro*. Upon *ex vivo* restimulation of CAR8 in the bone marrow, we found that, while IFNg production was highly variable, GZMB production was markedly greater in CAR8_MD_ (Figure 2E–F). CAR8_MD_ had significantly higher proportions of cells falling into short-lived effector cell (SLEC, IL7Ra−/KLRG1+) and effector memory precursor (EMP, CD27+/CD62L−) phenotypes, fewer cells in the central memory precursor phenotype (CMP, CD27+/CD62L+), and no change in memory precursor effector cell (MPEC, IL7Ra+/KLRG1−) populations (Figure S4B–E). Additionally, early expression of effector-associated TFs IRF4, T-bet and EOMES was greater in CAR8_MD_ (Figure 2G–I). Finally, while mice bearing WT high-antigen leukemia showed no survival difference after treatment with CAR8_MD_ versus CAR8_ND_, mice bearing CD19^Lo^ leukemia treated with CAR8_MD_ showed a significant survival benefit, with 20% of mice surviving to the 80 day experimental endpoint (Figure 2J). Together, these data show that CAR8_MD_ mediate superior clearance of CD19^Lo^ leukemia relative to CAR8_ND,_ associated with maintenance of effector function and expression of effector-associated markers.

### Treatment of leukemia-bearing mice with a low CAR+ cell dose reveals enhanced proliferative capacity and clearance of WT leukemia by naïve-derived CAR8.

**Figure 3:**
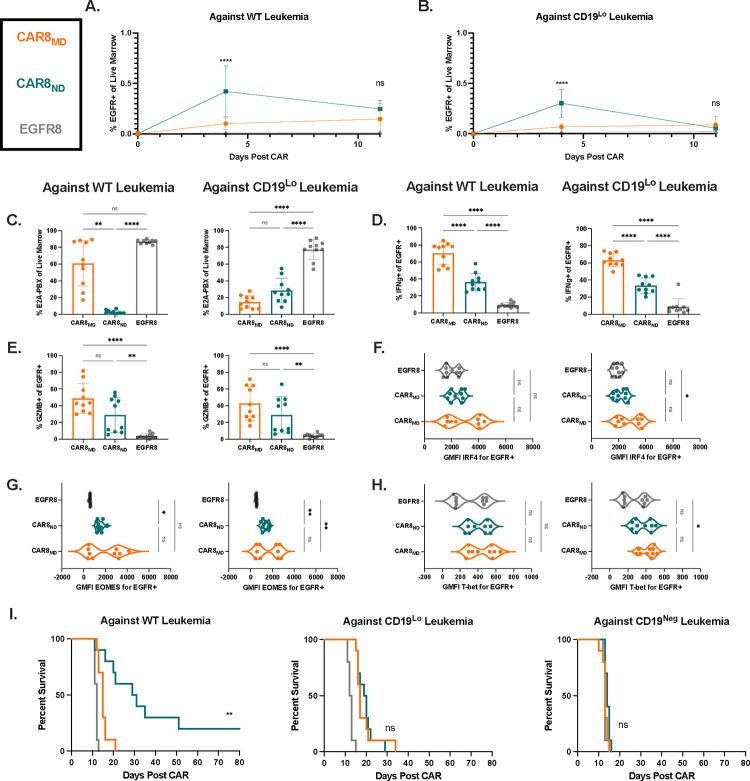
CAR8_ND_ exhibit enhanced expansion capacity and clearance of WT leukemia *in vivo* (low CAR dose). **3A-B:** Early T cell expansion (day +4) or persistence (day +11) after infusion of transduced T cells against WT leukemia **(A)** and CD19^Lo^ leukemia **(B)**. Transduced T cell populations measured by coexpression of CD8a+/TCRbeta+/EGFR+. **3C:** Clearance of WT and CD19^Lo^ leukemia at day +11 after CAR infusion. E2A-PBX measured by coexpression of B220+/CD22+. **3D-E:** Intracellular cytokine staining of interferon gamma **(D)** or granzyme B **(E)** in CAR T cells from whole bone marrow restimulated *ex vivo* with leukemia. Data represent mean +/− SD. **2F-H:** Intranuclear transcription factor staining of IRF4 **(F)**, EOMES **(G)**, or T-bet **(H)** on CAR+ T cells from mice bearing the indicated leukemia at day +4 after CAR infusion. Violin plot data represent median with quartiles. Data are from 2 pooled, independent experiments with n=10 mice per condition. * p<0.05, ** p<0.01, *** p<0.001, **** p<0.0001. **2I:** Survival of mice after treatment with 1e6 EGFR+ CAR or control T cells. * p<0.05, ** p<0.01, *** p<0.001, **** p<0.0001. Data are from 2 independent pooled experiments, total n=10 mice per group.

We next hypothesized that the benefit of enhanced proliferative capacity of naïve-derived CAR8 would emerge at a lower CAR+ cell dose (3e5). As anticipated, CAR8_ND_ expanded to significantly higher numbers in the bone marrow by day 4 regardless of leukemia antigen density, mirroring *in vitro* proliferative assays (Figure 3A–B, S3C, 1I–J). While CAR8_ND_ mediated enhanced clearance and survival in mice bearing WT leukemia, there was no improvement in leukemia clearance or survival of mice bearing CD19^Lo^ leukemia (Figure 3C, 3I, S3D), potentially due to reduced potency. Indeed, *ex vivo* IFNg production was greater in CAR8_MD_, although there was no difference in GZMB production or expression of IRF4, T-bet or EOMES (Figure 3D–H). CAR8_MD_ consistently demonstrated significantly higher proportions of SLECs at the early timepoint consistent with high CAR doses, but these differences disappeared by day 11 and no differences were seen in the MPEC population (Figure S5A,B). While EMP and CMP patterns mimicked high dose experiments, the differences were much less pronounced, indicating that naïve-derived cells largely became more “effector-like” with greater proliferative drive (Figure S5C,D), consistent with effector-polarization in the setting of low numbers of antigen-specific precursor populations^20, 21^. However, these changes, combined with the strong expansion, did not mediate survival benefit against CD19^Lo^ leukemia (Figure 3I). Finally, we predicted that at this lower cell dose, T cell dysfunction could emerge. Indeed, CAR8_MD_ expressed higher levels of exhaustion-associated markers against WT leukemia with failure of CAR8_MD_ to control leukemia (Figure S5E–F, I–J). Interestingly, we found that CD19^Lo^ leukemia drove similar proportions of exhaustion phenotypes in both CAR8 populations, demonstrating that chronic, uncleared antigen exposure, even at low antigen density, can drive dysfunction (Figure S5G–H, K–L). These findings highlight the importance of proliferative capacity and resistance to dysfunction afforded by CAR8_ND_ at limiting cell dose.

### Epigenetic profiling of naïve and memory-derived CAR8 shows differential chromatin accessibility at binding sites for bZIP, Tcf, Runx and other TF families.

**Figure 4:**
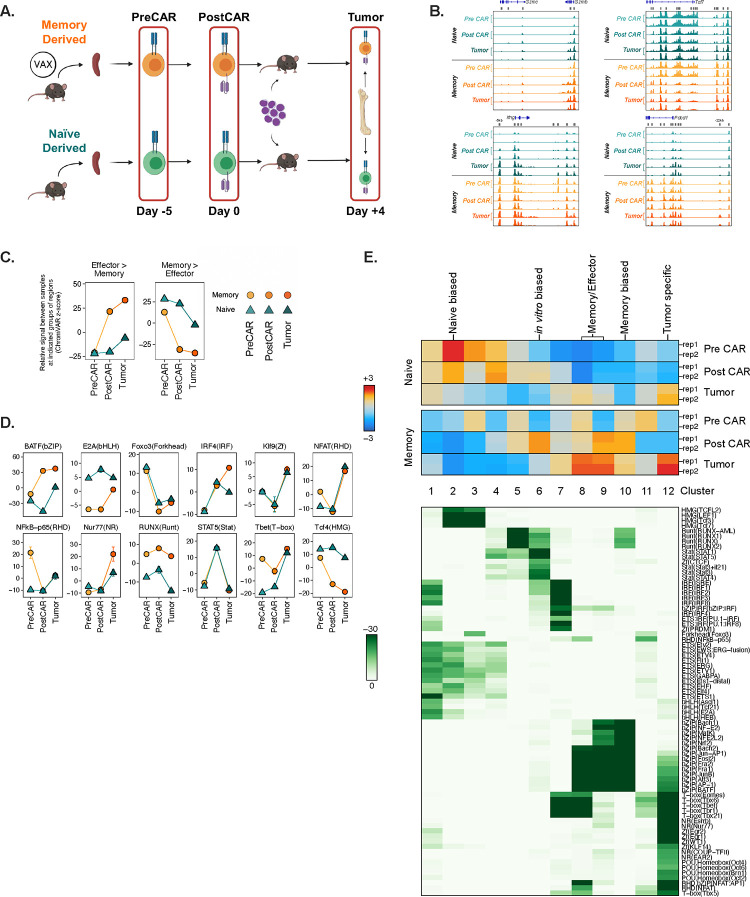
Prior antigen experience imprints chromatin accessibility states which follow unique patterns during CAR transduction and reinfusion. **4A:** Schematic: Layout for paired ATAC-seq/RNA-seq experiments. Memory-derived or naïve derived OT-I CD8+ T cells were sorted at three sequential timepoints: *Ex vivo* from donor mice before CAR transduction (“PreCAR”), *in vitro* after CAR transduction (“PostCAR”), and *ex vivo* after reinfusion into CD19^Lo^ leukemia-bearing *Rag1*^*−/−*^ mice (“Tumor”). **4B:** Chromatin accessibility at *Gzmb, Gzmc, Ifng, Tcf7 and Pdcd1* gene loci for naïve and memory-derived T cells at each timepoint. **4C:** ChromVAR deviation z-scores between indicated populations at differentially accessible regions between Effector and Memory T cells after LCMV-Armstrong infection^23^. Data are mean +/− range of two biological replicates. **4D:** Motif-associated ChromVAR deviation z-scores between indicated populations. Data are mean +/− range of two biological replicates **4E:** K-means clustering of relative ATAC-seq signal at differentially accessible regions (top, data from two biological replicates are shown) and motif enrichment in each cluster vs all regions (bottom).

We predicted that functional traits were a product of distinct epigenetic states, given that functional distinctions of naïve and memory-derived CAR8 were dictated by status prior to CAR transduction. To test this, we performed bulk ATAC-seq on naïve and memory-derived cells at three timepoints: *ex vivo* prior to CAR transduction (Day -5, “PreCAR”), *in vitro* after CAR transduction (Day 0, “PostCAR”), and after reinfusion into mice bearing CD19^Lo^ leukemia (Day 4, “Tumor”) (Figure 4A). Comparison of experimental replicates showed tight concordance of chromatin accessibility at each condition and timepoint (Figure S6A). Broadly, the data showed several thousand differentially accessible regions between either cell type compared to itself across timepoints, and between naïve and memory-derived cells at each timepoint (Figure S6B). We found predictable patterns of ATAC-seq signal at genetic loci involved in T cell activation or effector function, including higher accessibility in CAR8_MD_ at *Gzmb*, *Gzmc*, and the *Pdcd1* loci encoding for the PD1 protein. Concurrently, we found greater accessibility in CAR8_ND_ at the *Tcf7* loci encoding TCF1, a TF important for maintaining self-renewal capacity (Figure 4B).

We used ChromVAR^22^, to associate these changes in chromatin accessibility to previously defined datasets and potential TF activities. Based on relative chromatin accessibility at regions that were differentially accessible in a published comparison of effector and memory CD8+ T cells after acute viral infection with LCMV-Armstrong^23^, memory-derived CAR8 acquired effector-associated changes in chromatin accessibility during CAR generation in culture that were maintained after transfer into tumor bearing mice. CAR8 generated from memory T cells also had reduced chromatin accessibility at features associated with memory T cells. By comparison, naïve-derived CAR8 maintained chromatin accessibility patterns at regions associated with memory T cells and showed minimal skewing toward an effector-like profile^23^ (Figure 4C). To associate these changes with specific TF activities, we used ChromVAR to compare chromatin accessibility at regions containing DNA sequence motifs bound by different TFs (Figure 4D). Classifying this data using a kmeans clustering strategy, we found that there were distinct patterns of motif-associated chromatin accessibility between conditions and across each of the timepoints (Figure 4E). While motifs for bZIP and Irf family TFs broadly looked similar at the PreCAR timepoint, and became progressively enriched in memory cells, Tcf family motifs started similar and became enriched in naïve cells at the latter timepoints, while E2A family motifs started highly enriched in naïve and progressively converged. Uniquely, motifs for Runx family members were always more accessible in memory-derived cells and did not converge or diverge (Figure 4D–E, S6C). Overall, these data show epigenetic features imprinted in the starting CD8+ T cell population are maintained through CAR engineering.

### Prior antigen experience directs distinct transcriptomic patterns of naïve and memory-derived CAR8.

**Figure 5:**
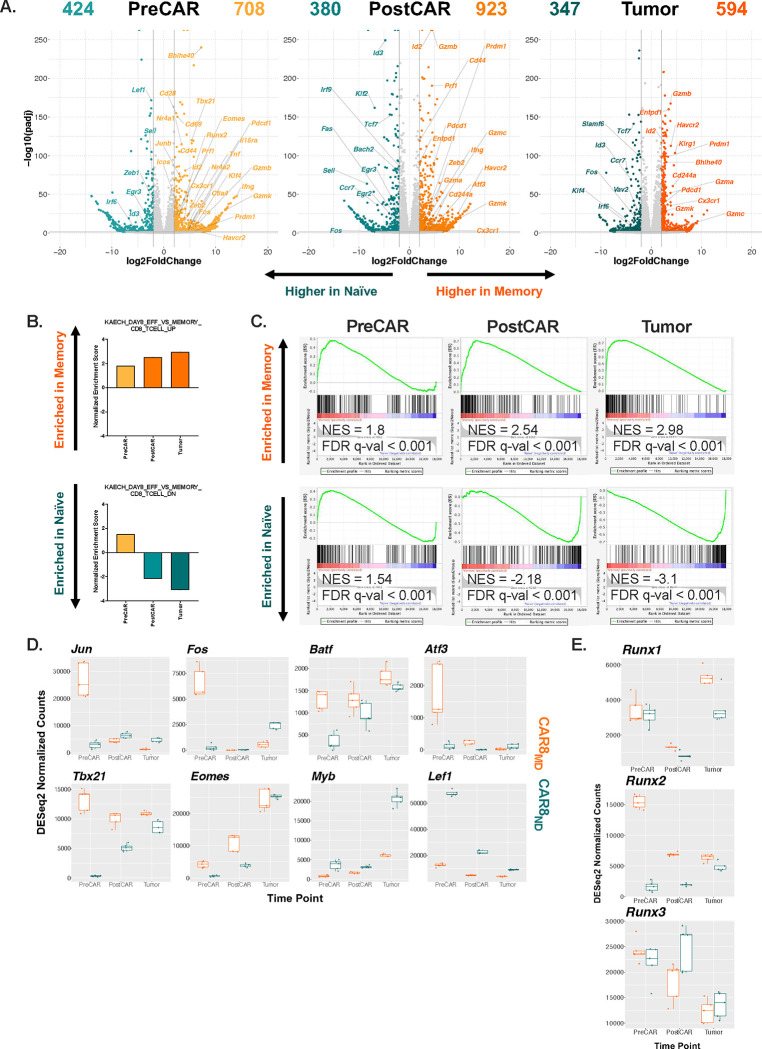
Prior antigen experience drives differential CAR8 transcriptomic states which follow unique patterns during CAR transduction and reinfusion. RNA-seq analysis was run on the timepoints/conditions indicated in the previous figure. **5A:** Volcano plots of significant differentially expressed genes between naïve and memory-derived cells at each of the three timepoints. **5B:** Normalized enrichment scores from gene set enrichment analysis (GSEA) of differentially enriched genesets between indicated CD8+ T cell subsets after LCMV-Armstrong acute viral infection^24^ **5C:** GSEA plots at each timepoint. **5D:** Top differentially expressed transcription factors at the “PreCAR” timepoint, generated using Ingenuity Pathway Analysis (IPA). **5E:** DESeq2-normalized counts of indicated transcription factors at each timepoint for naïve and memory-derived cells. **5F:** DESeq2-normalized counts of Runx family transcription factors at each timepoint for naïve and memory-derived cells. All statistics performed using DESeq2 with filtering threshold at 10, log2foldchange >2 and padj < 0.05.

To test whether the epigenetic states of naïve and memory-derived CAR8 resulted in concurrent transcriptomic changes, we performed bulk RNA-seq at the same timepoints as for ATAC-seq (Figure 4A). We found predictable differential gene expression at each timepoint, with genes associated with self-renewal and proliferative capacity (*Lef1, Sell, Id3, Tcf7, Slamf6, Il7r)* upregulated in the naïve-derived cells and genes associated with effector capacity and activation (*Prf1, Ifng, Klrg1, Gzmb, Prdm1, Id2, Pdcd1, Tbx21)* upregulated in the memory-derived cells (Figure 5A). Gene set enrichment analysis (GSEA) showed progressive bias by normalized enrichment score (NES) toward effector-like in memory-derived CAR8, and toward memory-like in naïve-derived CAR8^24, 25, 26^ (Figure 5B–C). Analysis with gene sets comparing memory and naïve T cells showed progressive decrease in the normalized enrichment score of memory or naïve-derived CAR8 toward the derivative cell population of each, suggesting the effector/memory gene set enrichment axis as the more accurate indicator of cell fate over time^24, 25^ (Figure S7A). Looking at the top differentially-expressed TFs between the populations at the PreCAR timepoint, we found many expected hits, including *Bhlhe40*, *Klf4*, *Tbx21*, *Id2* and many bZIP family members (*Jun*, *JunB*, *Fos*, *Cebpb*) represented in the memory-derived group, while *Zeb1*, *Myb* and *Lef1*, encoding TFs associated with self-renewal, were upregulated in the naïve-derived cells^23, 27^ (Figure S7D). Notably, among the Runx family, which showed uniquely stable differential motif accessibility between naïve and memory cells (Figure 4D), *Runx2* was among the most differentially expressed TF genes with marked overexpression in memory derived cells (Figure S7D). Ingenuity Pathway Analysis of global transcriptional profile implicated similar TF drivers^28^(Figure S7B) with numerous distinct patterns of differential TF expression between memory and naïve-derived T cells. However, a very common pattern among ChromVAR-implicated TFs was high initial expression in memory cells at the PreCAR timepoint, followed by a convergence in expression between memory and naïve-derived CAR T cells at the PostCAR and Tumor timepoints, as seen with bZIP family members *Jun*, *Fos* and *Atf3*, along with the gene *Tbx21*, encoding canonical effector TF T-bet (Figure 5D). Among the Runx family, *Runx1* and *Runx3* gene expression tracked relatively closely between memory and naïve-derived cells at each timepoint, while *Runx2* followed the “high in memory, then converging” pattern which was commonly found among other TF families (Figure 5E). In summary, naïve and memory-derived T cells show differential gene expression and gene set association with self-renewal or memory-associated genes and activation or effector-associated genes, respectively. Many relevant TF genes show a pattern of high initial expression in memory cells at the PreCAR timepoint which converges between the cell derivations upon transduction with a CAR and reinfusion into tumor-bearing hosts.

### RUNX2 overexpression boosts leukemia clearance, CAR T cell potency and CAR proportions in bone marrow.

**Figure 6:**
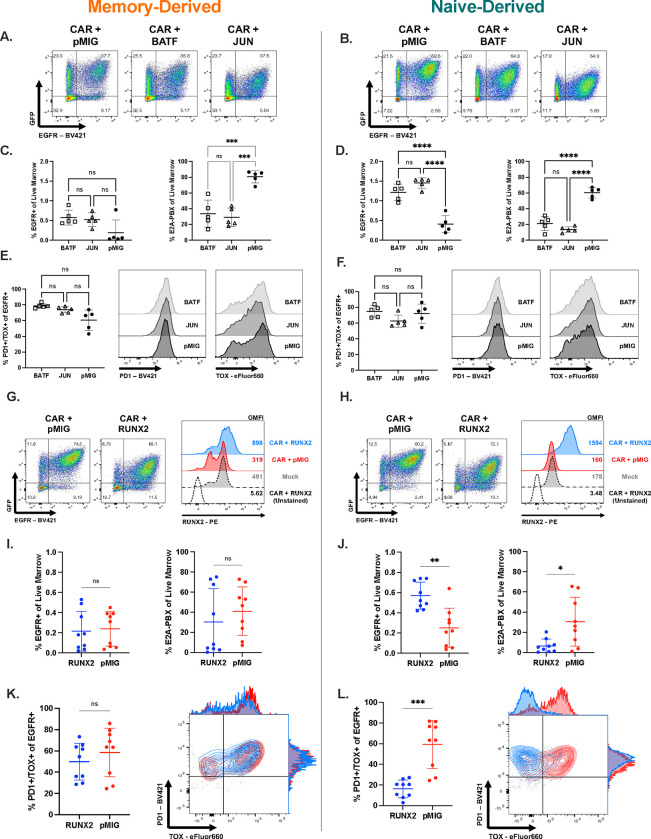
Runx2 overexpression as a novel strategy for enhancement of naïve-derived CD8+ CAR T cell potency and resistance to dysfunction. **6A-B:** Cotransduction of memory (A) or naïve (B) CD8+ T cells with CAR and pMIG-Empty, pMIG-BATF, or pMIG-JUN. For **6C-F & I-L**, *Rag1*^*−/−*^ mice were given leukemia on day −3, followed by 1e5 pMIG-Runx2 or pMIG-Empty co-transduced CAR8 on day 0. Bone marrow was analyzed by flow cytometry on day 11 post-CAR. **6C & D:** CAR T cell and leukemia proportions for naïve (C) and memory-derived (D) CAR T cells cotransduced with BATF, JUN or pMIG control. **6E & F:** Proportion of CAR T cells displaying PD1+/TOX+ phenotype. **6G-H:** Cotransduction of memory (G) or naïve (H) CD8+ T cells with CAR and pMIG-Empty or pMIG-Runx2 **and** intracellular staining for Runx2. **6I & J:** CAR T cell and leukemia proportions for naïve (C) and memory-derived (D) CAR T cells cotransduced with RUNX2 or pMIG control. **6K & L:** Proportion of CAR T cells displaying PD1+/TOX+ phenotype. Data in 6A,B,G & H are representative of 3–4 independent experiments. Data in 6C-F are from 1 experiment with n=5 mice per condition. Data in 6I-L are from 2 pooled, independent experiments with n=9 mice per condition. Data represent mean +/− SD. * p<0.05, ** p<0.01, *** p<0.001, **** p<0.0001.

To validate the epigenetic and transcriptomic data, we overexpressed two TFs from the ChromVAR-implicated bZIP family, BATF and c-Jun, both of which have been previously reported to impact CAR T cell function (Figure 6A–B)^29, 30, 31^. Although neither TF increased cytokine production or proliferation *in vitro* (Figure S8C–E), overexpression of either TF enhanced leukemia clearance by memory and naïve-derived CAR T cells (Figure 6C–D). There was no difference between BATF-CAR8 or JUN-CAR8 and control CAR8 in the PD1+ proportion (Figure S9C,E), co-expression of PD1 with markers of exhaustion (PD1+/CD39+ and PD1+/TOX+), or in the terminally exhausted Tcf1−/Tim3+ population (Figure S9D,F–H).

Due to the memory-like state of CAR8_ND_, we anticipated that comparison of factors enriched in memory cells over naïve cells could reveal important drivers of memory cell function that were not fully induced in naïve cells during the synthetic engineering process. Given the unique profile of chromatin accessibility for Runx-family binding motifs coupled with the pattern of *Runx2* transcript expression which was high in PreCAR memory CD8+ T cells and then lost upon CAR transduction, we hypothesized that establishing RUNX2 expression in CAR8_ND_ could enhance the existing memory-like profile of these T cells and boost T cell potency and anti-leukemia response. Murine RUNX2 was introduced into the pMSCV-IRES-eGFP (pMIG) backbone, containing a GFP reporter gene for long-term tracking of RUNX2-transduced T cell populations (RUNX2). Co-transduction of naïve CD8+ T cells with CAR-EGFR reporter and RUNX2-GFP reporter resulted in a large proportion of cells expressing both EGFR and GFP (Figure 6A). Upon intracellular staining for the RUNX2 protein, we found that the EGFR+ population in the RUNX2-transduced group showed approximately a 10-fold increase in RUNX2 expression relative to empty pMIG-transduced cells (Figure 6B). Co-culture of RUNX2-CAR8 and leukemia with a range of antigen densities revealed similar cytokine production and proliferation relative to pMIG-CAR8 (Figure 6C–D). To stress the ability of RUNX2-CAR8 to clear WT leukemia, we used an ultra-low CAR+ dose (1e5), against which both CAR8_ND_ and CAR8_MD_ exhibit markers of exhaustion and fail to control leukemia (Figure S7A–C). RUNX2 overexpression in CAR8_ND_ strongly enhanced leukemia clearance and increased CAR proportions and absolute numbers in the marrow at 11 days post-CAR infusion (Figure 6E–F). While there was no difference in the PD1+ proportion, consistent with similar activation, mice treated with RUNX2-CAR8_ND_ exhibited dramatically reduced proportion of PD1+/TOX+ cells, a lower proportion of PD1+/CD39+ cells and reduced proportions of TCF1−/TIM3+ cells, suggesting that RUNX2 overexpression counteracts the differentiation trajectory toward terminal exhaustion (Figure 6L, S9M–N,P)^27, 32^. CAR8_MD_ showed less of an increase in RUNX2 following transduction with RUNX2-eGFP (Figure S7F) potentially due to higher RUNX2 at baseline (Figure 5E). Nonetheless, RUNX2-overexpression resulted in a significant reduction in the PD1+/CD39+ exhaustion phenotype of RUNX2-CAR8_MD_ responding to WT leukemia and reduction in leukemia counts in marrow (Figure S9I) but no difference in other exhaustion phenotypes, CAR proportions or CAR counts (Figure 6K, S9K,L,O). We demonstrate that Runx2 overexpression in naïve-derived T cells enhances maintenance of CAR T cells in the marrow, boosts leukemia clearance and mediates a favorable exhaustion profile at a highly sub-curative CAR T cell dose with less impact in memory-derived CAR T cells, demonstrating that TF overexpression has a differential impact depending on starting T cell state.

## DISCUSSION

Factors underlying tumor relapse after CAR T cell therapy are a central focus of study in the field of cell therapies for leukemia. Advances have been made in understanding and engineering solutions to prevent tumor cell escape via antigen modulation, T cell dysfunction, and poor T cell trafficking/persistence^1^. However, defining *in vitro* and *in vivo* functional strengths and cellular profiles associated with different starting T cell populations may be an opportunity to specifically identify approaches to arm CAR T cells to overcome different tumor escape modalities. Importantly, refining qualities of the starting cell population will likely be a large contributor to efficacy of cellular therapeutics derived from healthy allogeneic donors or induced pluripotent stem cells, or in the case of *in vivo* transduction platforms targeting genetic payloads to specific cell populations. Recent work has sought to use targeted modulation of TFs to enhance CAR T cell function or prevent dysfunction, with several publications focusing on the bZIP TF family, including forced expression of BATF and c-Jun, or genetic deletion of the Nr4a family of nuclear receptors^29, 30, 31, 33, 34^. However, the impact of modulation of the bZIP family has been variable. Therefore, we set out to characterize functional attributes programmed by prior T cell antigen experience, with the prediction that these would be tied to epigenetic traits. We anticipated that downstream modulation of TFs implicated by this framework might have divergent functional outcomes depending on starting cell population.

In this study, we use a syngeneic murine model with anti-mouse CD19 CAR T cells targeting murine pre-B cell leukemia enabling more natural T cell differentiation trajectories without xenogeneic TCR stimulation. We also used a well-defined vaccine model for precise control of the antigen experience history of CAR T cells with a clonotypic TCR, with confirmation in a polyclonal memory response. With limiting T cell dose or low target antigen density as “stressors,” we report that antigen experience dictates multiple functional outputs of CAR T cells. Memory-derived CAR T cells exhibited stronger cytotoxic function across target antigen densities, while naïve-derived CAR T cells show greater proliferative capacity and more rapid cell cycle entry. This was associated with enhanced activity against low-antigen density leukemia by memory derived CAR T cells and enhanced activity of naïve-derived cells at limiting cell dose, a setting that drove phenotypic exhaustion and dysfunction of memory-derived cells.

T cell differentiation is a product of epigenetic and transcriptomic state^23, 27^ and while CAR T cells have been extensively profiled post-manufacturing, little work has been done to characterize effects of prior T cell state on post-transduction CAR T cell profiles^5^. We demonstrate that features of these states are maintained through CAR manufacturing and associate with differences in functional profiles. Specifically, we find significant differences in bZIP family transcription factors, which have been previously implicated in CAR T cell function^29, 30, 31^. BATF or JUN mediated enhanced leukemia clearance in our model independent of starting cell state, indicating that these TFs may derive most of their early *in vivo* activity via binding to NFAT-AP1 composite motifs, which show high accessibility in both cell types. Surprisingly, there was no difference in phenotypic exhaustion in BATF or JUN-overexpressing CAR T cells relative to control, indicating preservation of function in an exhausted state rather than prevention of exhaustion.

As a novel finding, we use epigenomic and transcriptomic assays and implicate modulation of Runx-family TFs, particularly Runx2, as having a likelihood for higher impact in naive-derived cells compared to memory. Ectopic RUNX2 expression in naïve-derived CAR T cells resulted in superior clearance of leukemia, higher proportions of cells in the marrow, and reduced proportions of cells displaying terminally exhausted phenotypes relative to control. Our data suggest that RUNX2 overexpression, in contrast to overexpression of bZIP family members, can enhance functional potency of naïve-derived CD8+ CAR T cells while preventing entry into the exhaustion differentiation trajectory.

In addition to their activity as transcriptional activators, Runx family members have been shown to recruit chromatin remodeling factors to Runx binding sites to open these sites and allow for transcriptional activation. In other model systems, RUNX2 has been shown to interact with SWI/SNF complexes, histone acetyltransferases (MOZ, p300), histone deacetylases (HDAC3, HDAC4, HDAC6) and histone methyltransferases (SUV39H1), along with all three TET family enzymes, indicating a plausibility for the ability for RUNX2 to recruit enzymes which participate in chromatin remodeling at RUNX binding motifs^35, 36, 37, 38^. These features could help explain the contribution of RUNX2 overexpression to the enhanced functionality and exhaustion resistance of CAR8_ND_ seen in our experiments. Additional studies will be necessary to fully elucidate the effects of RUNX2 in CAR T cells, and to confirm our findings in human CAR T cells. Nonetheless, using a model in which antigen history can be precisely controlled, we show that RUNX2 overexpression enhances *in vivo* CAR T cell function dependent on the starting T cell. Finally, we have generated a framework for the role of antigen experience on function of a CAR T cell in stress situations of limiting T cell dose or target antigen density and highlight the importance of considering this framework when assessing the impact of approaches to apply synthetic immunology to manipulate therapeutic immune effector cell functions.

## METHODS

See Supplemental Material.

## SUPPLEMENTAL METHODS

### Mouse Strains

B6.129S6-Rag2tm1Fwa Tg(TcraTcrb)1100Mjb (“OT-I,” Model #: 2334-F) mice were obtained from Taconic Biosciences. B6.SJL-Ptprca Pepcb/BoyJ (“PepBoy,” Strain #:002014), B6.129S7-Rag1tm1Mom/J (“*Rag1*^−/−^,” Strain #:002216), C57BL/6J mice (“B6,” Strain #:000664) were obtained from The Jackson Laboratory. Female mice were used for all experiments with B6 background mice. All mice were bred and/or maintained in the animal facility at University of Colorado Anschutz Medical Campus. All experiments were performed in compliance with the study protocol approved by University of Colorado Anschutz Medical Campus Institutional Animal Care and Use Committee (IACUC).

### Mouse CAR Constructs

The basic construction of the murine 1928z CAR was previously described^39^. The murine anti-CD19 scFv was Flag-tagged to enable CAR detection, and all ITAMs in the CD3zeta domain were kept intact. A truncated human EGFR reporter protein was incorporated following a 2A skip sequence to provide an additional method for detection of CAR-transduced cells^13^. The DNA was codon optimized, ordered from ThermoFisher GeneArt, and cloned into the MSCV-IRES-GFP backbone, a gift from Tannishtha Reya (Addgene plasmid # 20672 ; http://n2t.net/addgene:20672 ; RRID:Addgene_20672), using XhoI and ClaI enzyme sites. A control plasmid with just the truncated EGFR reporter in the MSCV backbone was generated using similar methods.

### Cell lines and media

E2A-PBX pre-B cell acute lymphoblastic leukemia was developed in the laboratory as previously described^14, 15, 16^. Murine T cells and leukemia were cultured in Complete Mouse Media (CMM), consisting of RPMI 1640 medium (Gibco) with 10% heat-inactivated fetal calf serum (Omega Bio), 1% nonessential amino acids (Gibco), 1% sodium pyruvate (Gibco), 1% penicillin/streptomycin (Gibco), 1% L-glutamine (Gibco), 1% HEPES buffer (Gibco) and 50uM 2-mercaptoethanol (Sigma-Aldrich).

### Mouse CAR Transduction

CAR transduction was performed as previously described^14, 15, 16^. Briefly, spleens from 6–10 week old donor mice were harvested and CD8+ T cells were isolated using EasySep Mouse CD8+ T cell Isolation Kit from STEMCell Technologies or bulk T cells were isolated using the Mouse CD3+ T Cell Enrichment Column Kit (R&D Biosciences, Cat No. MTCC-25). On day 1, T cells were activated on anti-CD3/anti-CD28 Mouse T cell Activator DynaBeads (Invitrogen) at a 1:1 cell:bead ratio and cultured at 1e6/mL in CMM in the presence of rhIL-2 (40IU/mL) and rhIL-7(10ng/mL) from R&D Systems. On days 2 and 3, retroviral supernatant was added to Retronectin-coated (Takara Biosciences) 6 well plates and spun at 2000×g and 32°C for 2–3 hours. Supernatant was then removed and activated T cells were added to the wells at 1.67mL/well. On day 4, beads were removed and T cells were resuspended at 1e6/mL in fresh media with cytokines. CAR transduction was determined post-debeading by analyzing T cells by flow cytometry for a FLAG/EGFR double-positive population (or EGFR single-positive for control T cells), and T cells were used in assays or infused into mice on day 5 or 6.

### Vaccine Model

The ovalbumin vaccine consists of 100ug whole ovalbumin protein (InvivoGen, Cat. code: vac-pova-100), 40ug anti-mouse CD40 (BioXCell, Catalog #BE0016–2) and 40ug Polyinosinic:polycytidylic acid [Poly (I:C)] (InvivoGen, Cat. code: tlrl-pic-5) per mouse, resuspended to 200uL total volume in PBS^17, 18, 19^. CD8+ T cells were isolated from naïve 6 to 8 week old OT-I mouse splenocytes using the Mouse CD3+ T Cell Enrichment Column Kit (R&D Biosciences, Cat No. MTCC-25). PepBoy mice were given 5e3 OT-I T cells retro-orbitally and concurrently vaccinated intravenously. 3–4 weeks later, spleens from 5–20 vaccinated mice were pooled and CD45.2+ OT-I memory T cells were isolated using the EasySep Mouse CD8+ T cell Isolation Kit, followed by column isolation using biotinylated anti-mouse CD45.2 (BioLegend, Cat # 109804), LS Columns (Miltenyi Biotec, Order No. 130-042-401), and anti-Biotin MicroBeads (Miltenyi Biotec, Order No. 130-;090-485). Naïve T cells from 1–5 naïve OT-I donors were isolated in parallel. T cells were then activated and transduced as described for downstream experiments.

### Generation of CD19^Lo^ E2A-PBX leukemia cell lines

The E2A-PBX murine leukemia was generated in our lab as previously described ^14^. CD19 knockout leukemia was produced using CRISPR/Cas9. A previously-validated murine CD19-targeting sgRNA^15^ from Integrated DNA Technologies was incubated with recombinant Cas9 from TakaraBio (Cat# 632641) to create an RNP complex. RNP was then electroporated into E2A-PBX using the Lonza 4D-Nucleofector X with nucleofector solution SG and pulse program CM-147. Electroporated cells were allowed to recover for 48 hours and then FACS-sorted twice to obtain a pure CD19 knockout cell line. This cell population was additionally single cell cloned to create a CD19 knockout single cell clone prior to transduction with murine CD19. A truncated/non-signaling murine CD19 was cloned into the pLV.SP146.gp91.GP91.cHS4 plasmid, a gift from Didier Trono (Addgene plasmid # 30480 ; http://n2t.net/addgene:30480 ; RRID:Addgene_30480). Backbones were generated with the hEF1a promoter (pLV.hEF1a.cHS4) or the hUbC promoter (pLV.hUbC.cHS4) from the pLenti6/UbC/mSlc7a1 plasmid, a gift from Shinya Yamanaka (Addgene plasmid # 17224 ; http://n2t.net/addgene:17224 ; RRID:Addgene_17224). VSV-G pseudotyped lentivirus was generated as described and E2A-PBX CD19KO underwent a single round of transduction using standard protocols, followed by single cell cloning to obtain clonally-derived lines expressing defined levels of CD19 target antigen.

### Flow Cytometry

Flow cytometry analysis was performed using an LSR-Fortessa X-20 flow cytometer (BD Biosciences) and analyzed using FlowJo (BD Biosciences). Monoclonal antibodies used in staining are listed in the supplemental methods. Intracellular flow cytometry staining was performed using the TrueNuclear Transcription Factor Buffer Set (BioLegend) for *ex vivo* staining of transcription factors, Cytofix/Cytoperm Fixation/Permeablization Kit (BD Biosciences) for intracellular cytokine staining, and Mouse Foxp3 Buffer Set (BD Biosciences) for intracellular staining of Ki67 and Runx2.

### CD107a Degranulation, Intracellular Cytokine Staining (ICCS), Ki67 and CellTrace Dilution *In Vitro* Assays

*In vitro* assays were performed using a 1:1 effector to target cell ratio with 1e5 of each cell type in a 96-well round-bottom plate followed by analysis by flow cytometry at the indicated timepoints. Degranulation assays were performed by incubation for 4 hours in the presence of 2uM monensin and 1uL of CD107a antibody. ICCS was performed by incubation for 6 hours, with 1uM monensin and 2.5uM Brefeldin A added at 1 hour in. Ki67 was performed by incubation for 18 hours, followed by intracellular staining for Ki67. CellTrace dilution assays were performed by staining T cells with CellTrace Violet (Thermo Fisher Scientific) per manufacturer protocols followed by incubation with target cells for 72 hours.

### LCMV infection and T cell isolation

6 week old female C57BL/6 mice were injected retro-orbitally with 2e5 PFU of LCMV-Armstrong. 4 weeks later, CD8+ T cells were isolated from 5 pooled spleens using the EasySep Mouse CD8+ T cell Isolation Kit from STEMCell Technologies and then FACS-sorted to obtain Memory (CD8+/CD44+/CD49d^hi^) and Naïve (CD8+/CD44−/CD49d^lo^/CD62L+) populations from the same mice. T cells were then transduced using the standard transduction protocol as described.

### *In vivo* experiments in *Rag1*^−/−^ hosts

Experiments were carried out using a timeline previously optimized in the lab^14^. Briefly, *Rag1*^−/−^ hosts were inoculated with 1e6 E2A-PBX by tail vein I.V. injection on day −3 followed by CAR T cells via retroorbital injection at either 1e5, 3e5 or 1e6 CAR+ cell dose on day 0. Bone marrow was harvested and analyzed by flow cytometry on day 4 or 11 post-CAR infusion, or mice were euthanized at humane endpoints for survival experiments. *Ex vivo* stimulation for cytokine production was performed using 1e6 E2A-PBX WT to stimulate approximately 1.5e6 whole bone marrow cells from each individual mouse, with pooled bone marrow from each n=5 experimental group stimulated by E2A-PBX CD19^Neg^ as a negative control. Cells were co-cultured for 6 hours, with 1uM monensin and 2.5uM Brefeldin A added at 1 hour in and then analyzed by flow for cytokine production.

### Bulk ATAC and RNA sequencing experimental setup and workflows

OT-I CD8+ T cells were isolated from vaccinated or naïve donors and CARs were transduced into T cells as described above. CAR8 *Rag1*^−*/*−^ hosts were inoculated with 1e6 E2A-PBX CD19^10,000^ followed by 1e6 CAR8_MD_ or CAR8_ND_ on the timeline described above. At day 4 post-CAR infusion, bone marrow from 10 mice per CAR group was harvested and pooled. At each of 3 timepoints, CD8+ cells were isolated using the EasySep Mouse CD8+ T cell Isolation Kit from STEMCell Technologies and then FACS-sorted to obtain 50,000 cells per condition. ATAC-seq and RNA-seq were performed in triplicate on separate sorted aliquots of 50,000 cells at “Pre-CAR/Day −5” (*ex vivo,* directly after isolation of memory or naïve CD8+ T cells from donor mice), “Post-CAR/Day 0” (*in vitro*, after CAR manufacturing) and “Tumor/Day 4” (*ex vivo*, after reinfusion into leukemia bearing mice). Experimental analyses were performed on the first technical replicate from 2 separate experimental replicates. For RNA-seq, cells were homogenized in QIAzol Lysis Reagent (Qiagen, Cat. No. 79306) and then frozen at −80C for processing within 2 weeks. Samples were thawed and processed using the miRNeasy Micro Kit (Qiagen, Mat. No. 1071023), with on-column DNase treatment (RNase-Free DNase Set, Qiagen, Cat. No. 79254), both according to manufacturer protocols. RNA purity, quantity and integrity was determined with NanoDrop (ThermoFisher Scientific) and TapeStation 4200 (Agilent) analysis prior to RNA-seq library preparation. The Universal Plus mRNA-Seq library preparation kit with NuQuant was used (Tecan) with an input of 200ng of total RNA to generate RNA-seq libraries. Paired-end sequencing reads of 150bp were generated on NovaSeq 6000 (Illumina) sequencer at a target depth of 40 million clusters/80 million paired-end reads per sample. Raw sequencing reads were de-multiplexed using bcl2fastq. For ATAC-seq, cells were immediately processed using the Omni-ATAC protocol as previously described^40^. Briefly, sorted cells were washed once in 1X PBS, lysed, washed once in Wash Buffer and then the transposition reaction was carried out at 32°C for 30 minutes on a thermomixer set to 1000 rpm. Transposed chromatin was then purified using the Zymo Clean and Concentrator 5 Kit (Zymo Research, Cat # D4013) using manufacturer protocols. DNA was then ran on PCR for 12 total cycles with matched barcoding primers^41^. PCR reactions were then size-selected using AMPure XP beads (Beckman Coulter Life Sciences, Product No: A63880) and checked for quality and size distribution using TapeStation 4200 with D5000 reagents (Agilent). Libraries were pooled at equimolar ratios for sequencing and paired-end sequencing reads of 150bp for the first replicate and 50bp for the second replicate were generated on NovaSeq 6000 (Illumina) sequencer at a target depth of 40 million clusters/80 million paired-end reads per sample. Raw sequencing reads for replicate 1 were shortened to match the read lengths for replicate 2 using trimmomatic function CROP. Raw sequencing reads were de-multiplexed using bcl2fastq.

### RNA-seq Data Analysis

Quality of fastq files was accessed using FastQC (v.0.11.8) (http://www.bioinformatics.babraham.ac.uk/projects/fastqc), FastQ Screen (v.0.13.0)^42^ and MultiQC (v.1.8)^43^. Illumina adapters and low-quality reads were filtered out using BBDuk (v. 38.87) (http://jgi.doe.gov/data-and-tools/bb-tools). Trimmed fastqc files were aligned to the mm10 murine reference genome and aligned counts per gene were quantified using STAR (v.2.7.9a) ^44^. Differential gene expression analysis was performed using the DESeq2 package^45^. Pathway enrichment analysis was performed using GSEA (UC San Diego/Broad Institute)^26, 46^, Metascape^47^ for gene mapping and IPA (Qiagen)^28, 48^. Differential gene expression was plotted using GraphPad Prism or ggplot2 (R package). RNA-seq differential gene expression statistics were run using the DESeq2 R package, with filtering threshold at 10 with greater than 2-fold change and adjusted p value < 0.05.

### ATAC-seq Data Analysis

Fastq files were used to map to the mm10 genome using the ENCODE ATAC-seq pipeline (https://www.encodeproject.org/atac-seq/), with default parameters, except bam files used for peak calling were randomly downsampled to a maximum of 50 million mapped reads. Peaks with a MACS2(https://pypi.org/project/MACS2/) computed q value of less than 1e-6 and a signalValue of more than 4 in at least one replicate were merged with bedtools^49^ function intersect and processed to uniform peaks with the functions getPeaks and resize from R package ChromVAR^22^. Reads overlapping peaks were enumerated with getCounts function from ChromVAR and normalized and log2-transformed with voom from R package limma^50^. Peaks with 3 or more normalized counts per million mapped reads at least one replicate were included to define a global peak set of 82,410 peaks. Pairwise Euclidean distances were computed between all samples using log2-transformed counts per million mapped reads among the global peak set. Differentially accessible peaks were identified in pairwise comparisons based on fdr adjusted p values of less than 0.01, fold change of at least 4 and with an average of 3 normalized counts per million mapped reads using R package limma. Motif associated variability in ATAC-seq signal was computed with R package ChromVAR. Genome-wide visualization of ATAC-seq coverage was computed with deeptools^51^ function coveragebam, using manually computed scale factors based on the number of reads within the total peak set.

### Statistics

Statistical tests for all experiments except sequencing analyses were performed using GraphPad Prism v9.0 for Macintosh (GraphPad Software). Comparisons between three groups were made with ordinary one-way ANOVA with Holm-Sidak’s multiple comparisons test, Brown-Forsythe and Welch one-way ANOVA with Dunnett’s T3 multiple comparisons test, or Kruskal-Wallis non-parametric test with Dunn’s multiple comparisons test were used depending on variance in standard deviations. Two-way ANOVA or mixed effects analysis with Tukey’s multiple comparisons test was used for *in vitro* experimental comparisons with multiple antigen densities and *in vivo* CAR expansion data. Two-tailed ordinary t test, Welch’s t test or Mann-Whitney test were performed for comparisons with two groups depending on normality of distributions. For multiple comparisons of two groups, multiple unpaired t tests or multiple Welch’s t tests, both with Holm-Sidak’s multiple comparisons test, were performed when appropriate depending on variance in standard deviations. Log-rank (Mantel-Cox) test was used for survival curve comparisons. All data represented as mean +/− standard deviation. * p<0.05, ** p<0.01, *** p<0.001, **** p<0.0001. Technical and experimental replicates in each dataset are indicated in figure legends.

## Supplementary Material

Supplement 1

## Data Availability

All data is readily available from authors upon request or accessible at Gene Expression Omnibus (**GEO Accession Number will be provided before paper acceptance**). All materials are either commercially available as described or available from authors upon request.
