# Supplementary material for: Antigen experience history directs distinct functional states of CD8+ CAR T cells during the anti-leukemia response
Source: Res Sq. 2023 Dec 21:rs.3.rs-3712137. Preprint. [Version 1] doi: 10.21203/rs.3.rs-3712137/v1 (PMC10775394; doi:10.21203/rs.3.rs-3712137/v1)
Supplement: Supplement 1 [file NIHPPrs3712137v1-supplement-1.pdf]

Figure S1

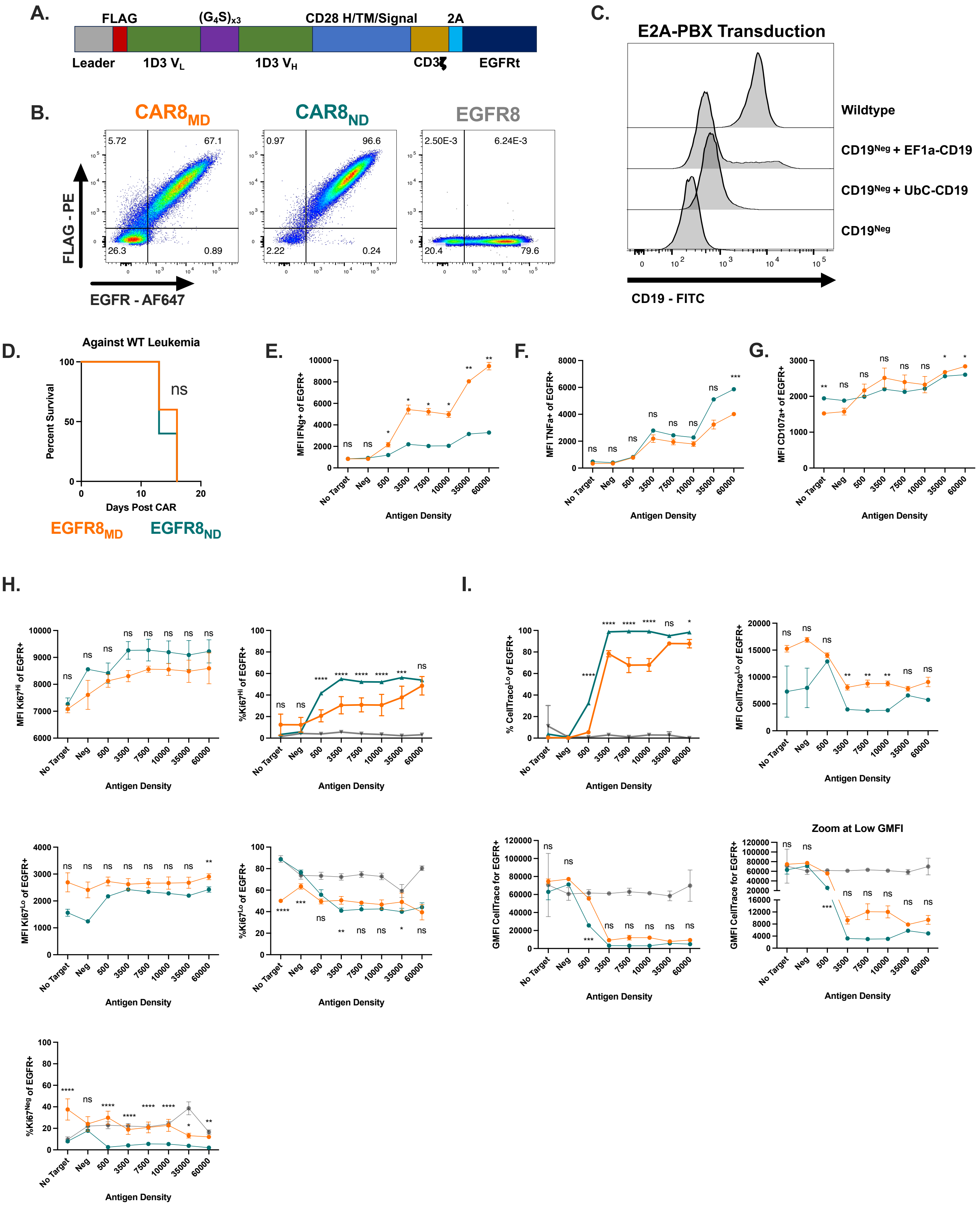
A.
FLAG
(G4S)x3
CD28 H/TM/Signal
2A
Leader
1D3 VL
1D3 VH
CD3ζ
EGFRt
B.
CAR8MD
CAR8ND
EGFR8
5.72
67.1
26.3
0.89
0.97
96.6
2.22
0.24
2.50E-3
6.24E-3
20.4
79.6
FLAG - PE
EGFR - AF647
C.
E2A-PBX Transduction
Wildtype
CD19Neg + EF1a-CD19
CD19Neg + UbC-CD19
CD19Neg
CD19 - FITC
D.
Against WT Leukemia
Percent Survival
Days Post CAR
ns
EGFR8MD
EGFR8ND
E.
MFI IFNg+ of EGFR+
F.
MFI TNFa+ of EGFR+
G.
MFI CD107a+ of EGFR+
Antigen Density
No Target
Neg
500
3500
7500
10000
35000
60000
H.
MFI Ki67Hi of EGFR+
%Ki67Hi of EGFR+
MFI Ki67Lo of EGFR+
%Ki67Lo of EGFR+
%Ki67Neg of EGFR+
I.
% CellTraceLo of EGFR+
MFI CellTraceLo of EGFR+
GMFI CellTrace for EGFR+
Zoom at Low GMFI

Figure S1: E2A-PBX/mCD19 antigen density model and murine anti-CD19 CAR T cells, and additional statistical comparisons of *in vitro* data (Related to Figure 1)

S1A: Schematic of the anti-mouse CD19 CAR contained in pMSCV backbone. **S1B:** Coexpression of CAR and EGFR on murine CAR T cells. **S1C:** Engineering of murine leukemia with lentiviral vectors containing hUbC or hEF1a promoters driving the CD19 transgene. **S1D:** Survival of mice after treatment with 1e6 EGFR+ (EGFR8, non-CAR expressing) naïve or memory-derived CD8+ T cells. Data is from 1 experiment, total n=5 mice per group. **S1E:** Mean fluorescence intensity of IFNg+ cell population. **S1F:** Mean fluorescence intensity of TNFa+ cell population. **S1G:** Mean fluorescence intensity of CD107a+ population. **S1H:** Statistical comparisons of Ki67^{Neg} (% Ki67Neg of EGFR+), Ki67^{Lo}(%Ki67^{Lo} of EGFR+, MFI Ki67^{Lo} of EGFR+) and Ki67^{Hi}(%Ki67^{Hi} of EGFR+, MFI Ki67^{Hi} of EGFR+) populations . **S1I:** Statistical comparisons of CellTrace^{Lo} (% CellTrace^{Lo} of EGFR+, MFI CellTrace^{Lo} of EGFR+) and total EGFR+ (GFMI CellTrace, GFMI CellTrace with zoomed axis) populations. Data represent mean +/- SD. * p<0.05, ** p<0.01, *** p<0.001, **** p<0.0001.

Figure S2

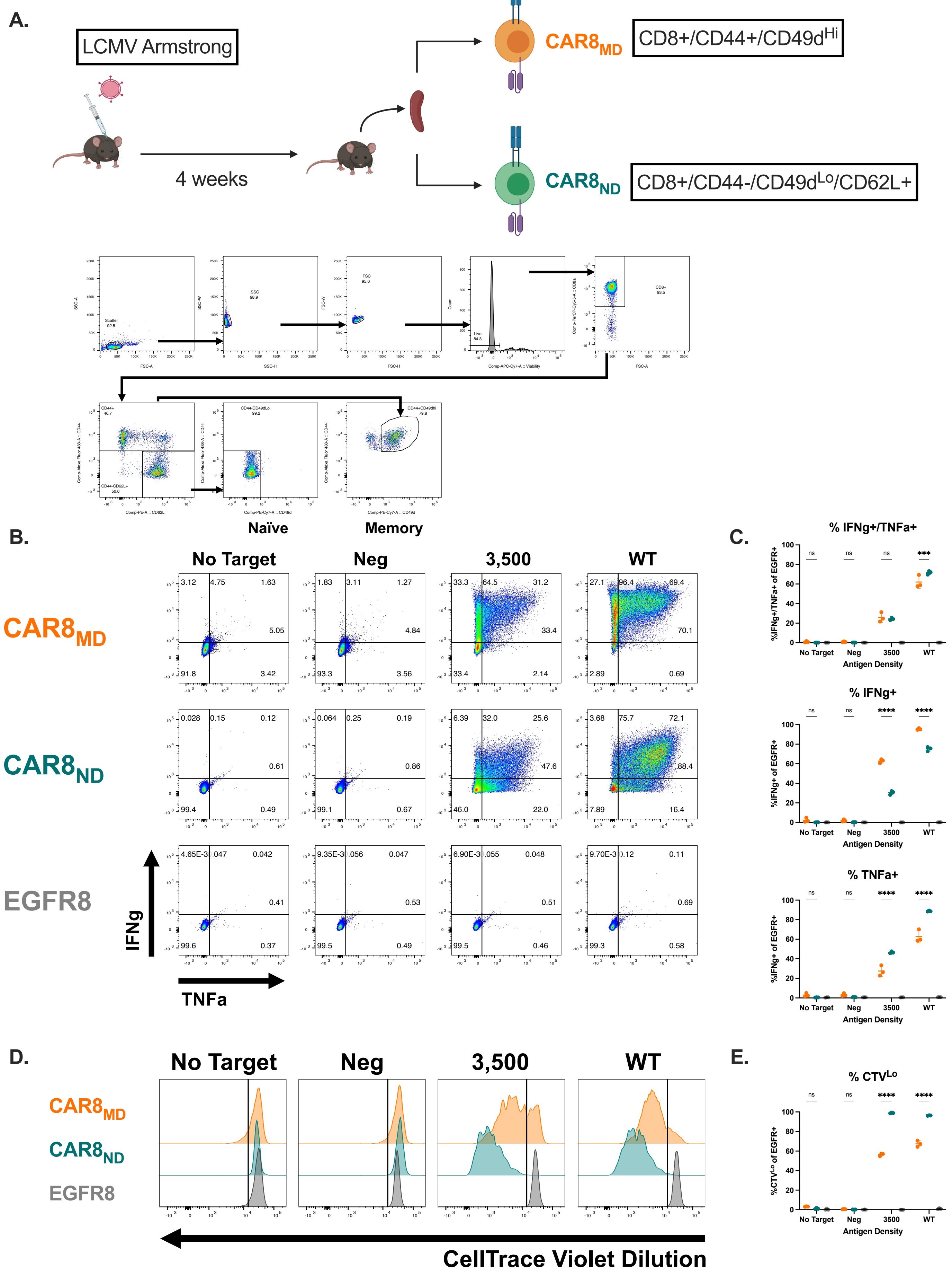
A.
LCMV Armstrong
4 weeks
CAR8MD
CD8+/CD44+/CD49dHi
CAR8ND
CD8+/CD44-/CD49dLo/CD62L+
Naïve
Memory
B.
No Target
Neg
3,500
WT
CAR8MD
CAR8ND
EGFR8
IFNg
TNFa
C.
% IFNg+/TNFa+
% IFNg+
% TNFa+
Antigen Density
D.
No Target
Neg
3,500
WT
CAR8MD
CAR8ND
EGFR8
CellTrace Violet Dilution
E.
% CTVLo
Antigen Density

Figure S2: Polyclonal pathogen-elicited $CAR8_{MD}$ function similarly to vaccine-elicited $CAR8_{MD}$ (Related to Figure 1).

S3A: Schematic: LCMV model for generating memory CD8+ T cells. C57BL/6 hosts were infected with LCMV-Armstrong. 4 weeks later, naïve and memory CD8+ T cells were sorted from the same hosts using the indicated FACS markers and used to manufacture $CAR8_{MD,}$ memory-derived or $CAR8_{ND}$, naïve-derived or EGFR8 control cells. **S3B:** Intracellular cytokine staining of IFNg and TNFa after 6 hour co-culture assay. **S3C:** Quantifications of proportions of IFNg+ and TNFa+ cells of EGFR+ population. **S3D:** Proliferation as measured by dilution of CellTrace Violet dye after 72 hour co-culture assay. **S3E:** Quantification of CellTrace assay, proportions of $CellTrace^{Lo}$ cells. All assays were performed with n=3 technical replicates, and are representative of 2 independent experiments. Data represent mean +/- SD. * p<0.05, ** p<0.01, *** p<0.001, **** p<0.0001.

Figure S3

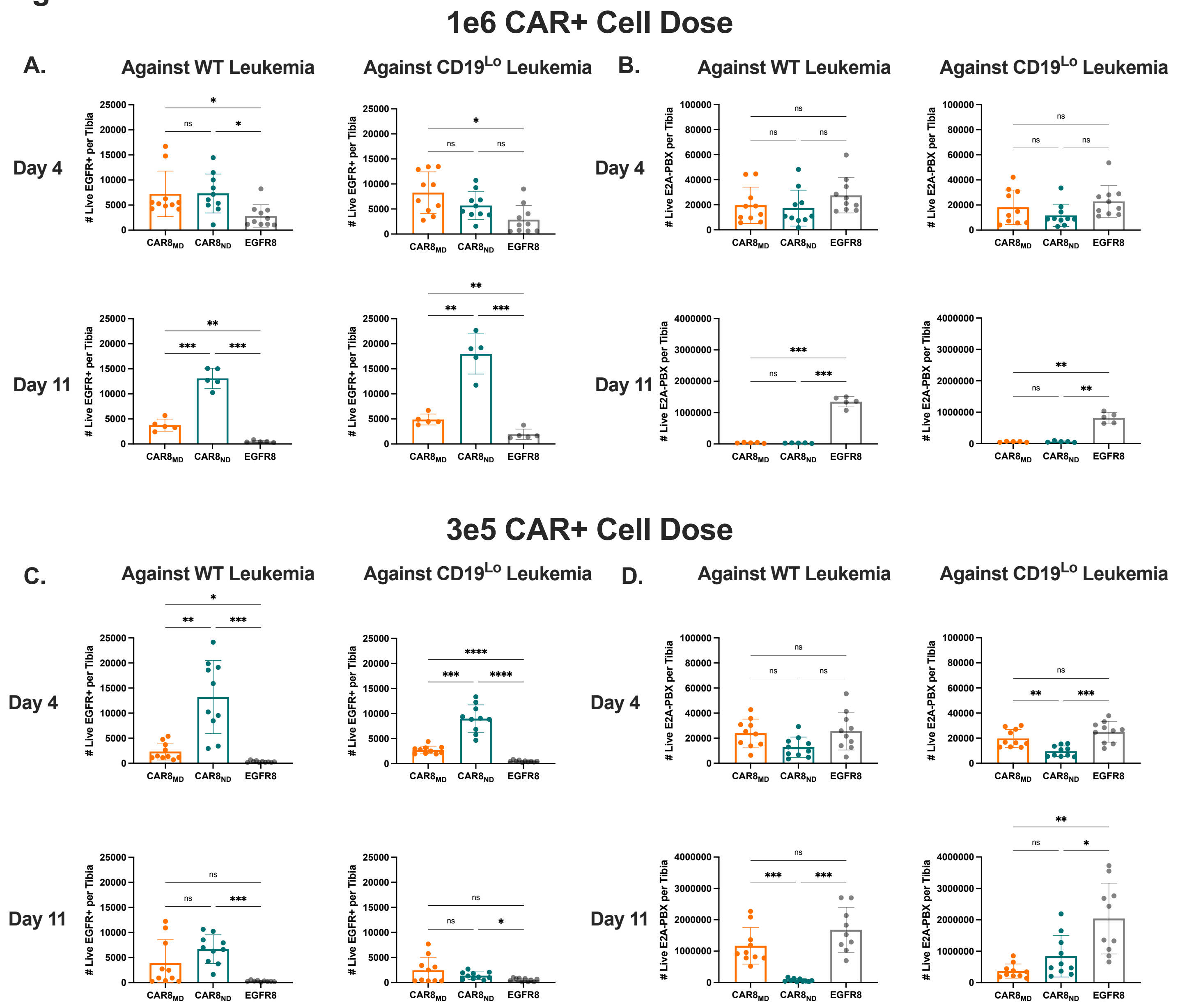

Figure S3: CAR T cells and leukemia counts per tibia for *in vivo* data (Related to Figures 2 & 3)

All analyses in this figure are done on the same experiments described in Figures 2 and 3. Counts data was generated by flushing a single tibia and using total tibia counts and cytometer proportions data to calculate CAR and leukemia cell counts per tibia. **S8A:** CAR counts for 1e6 CAR dose experiments. **S8B:** Leukemia counts for 1e6 CAR dose experiments. **S8C:** CAR counts for 3e5 CAR dose experiments. **S8D:** Leukemia counts for 3e5 CAR dose experiments. Data are from 2 pooled, independent experiments with n=10 mice per condition, apart from the 1e6 CAR dose day 11 timepoint, which contains data from one experiment with n=5 mice per condition. Data represent mean +/- SD. * $p<0.05$, ** $p<0.01$, *** $p<0.001$, **** $p<0.0001$.

Figure S4

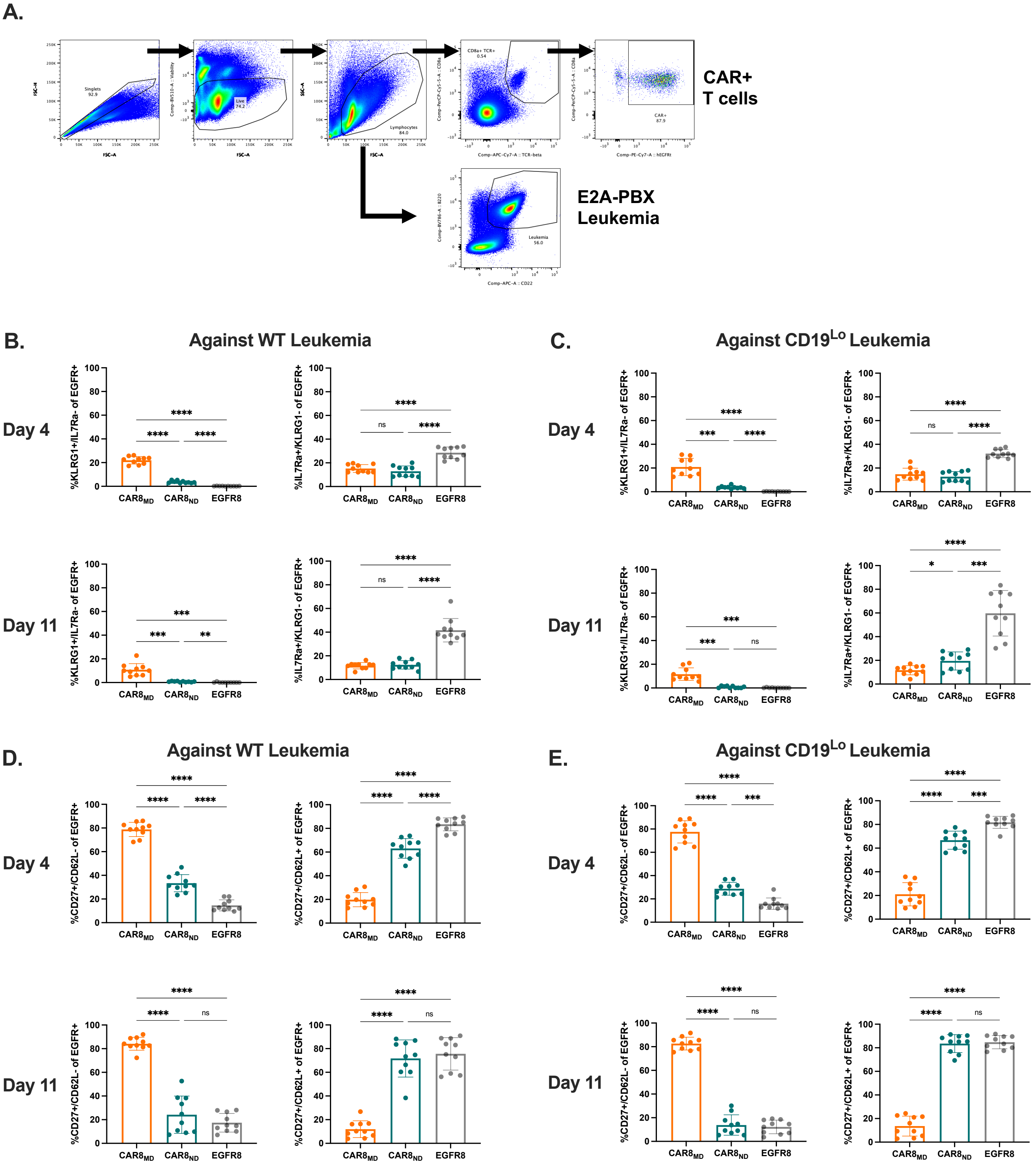
A.
CAR+ T cells
E2A-PBX Leukemia
B.
Against WT Leukemia
C.
Against CD19Lo Leukemia
Day 4
Day 11
%KLRG1+/IL7Ra- of EGFR+
%IL7Ra+/KLRG1- of EGFR+
CAR8MD
CAR8ND
EGFR8
D.
Against WT Leukemia
E.
Against CD19Lo Leukemia
%CD27+/CD62L- of EGFR+
%CD27+/CD62L+ of EGFR+
ns

Figure S4: Basic characterization of *in vivo* model and additional *in vivo* effector/memory phenotyping at high CAR dose (Related to Figure 2).

S4A: Basic flow cytometry gating strategy for *in vivo* experiments. Total events were gated by Singlets, Live Cells and then Lymphocytes, followed by CD8a+/TCRbeta+/EGFR+ cells for CAR8/EGFR8 or B220+/CD22+ cells for E2A-PBX. S4C-F are from experiments with the 1e6 EGFR+ cell dose. **S4B:** Proportions of CAR8 with the short-lived effector cell (SLEC, IL7Ra-/KLRG1+) or memory precursor effector cell (MPEC, IL7Ra+/KLRG1-) phenotypes at the indicated timepoint against WT leukemia. **S4C:** Proportions of CAR8 with the short-lived effector cell (SLEC, IL7Ra-/KLRG1+) or memory precursor effector cell (MPEC, IL7Ra+/KLRG1-) phenotypes at the indicated timepoint against $CD19^{Lo}$ leukemia. **S4D:** Proportions of CAR8 with the effector memory precursor (EMP, CD27+/CD62L-) or central memory precursor (CMP, CD27+/CD62L+) phenotypes at the indicated timepoint against WT leukemia. **S4E:** Proportions of CAR8 with the effector memory precursor (EMP, CD27+/CD62L-) or central memory precursor (CMP, CD27+/CD62L+) phenotypes at the indicated timepoint against $CD19^{Lo}$ leukemia. Data in S4C-F are from 2 pooled, independent experiments with n=10 mice per condition. Data represent mean +/- SD. * $p<0.05$, ** $p<0.01$, *** $p<0.001$, **** $p<0.0001$.

Figure S5

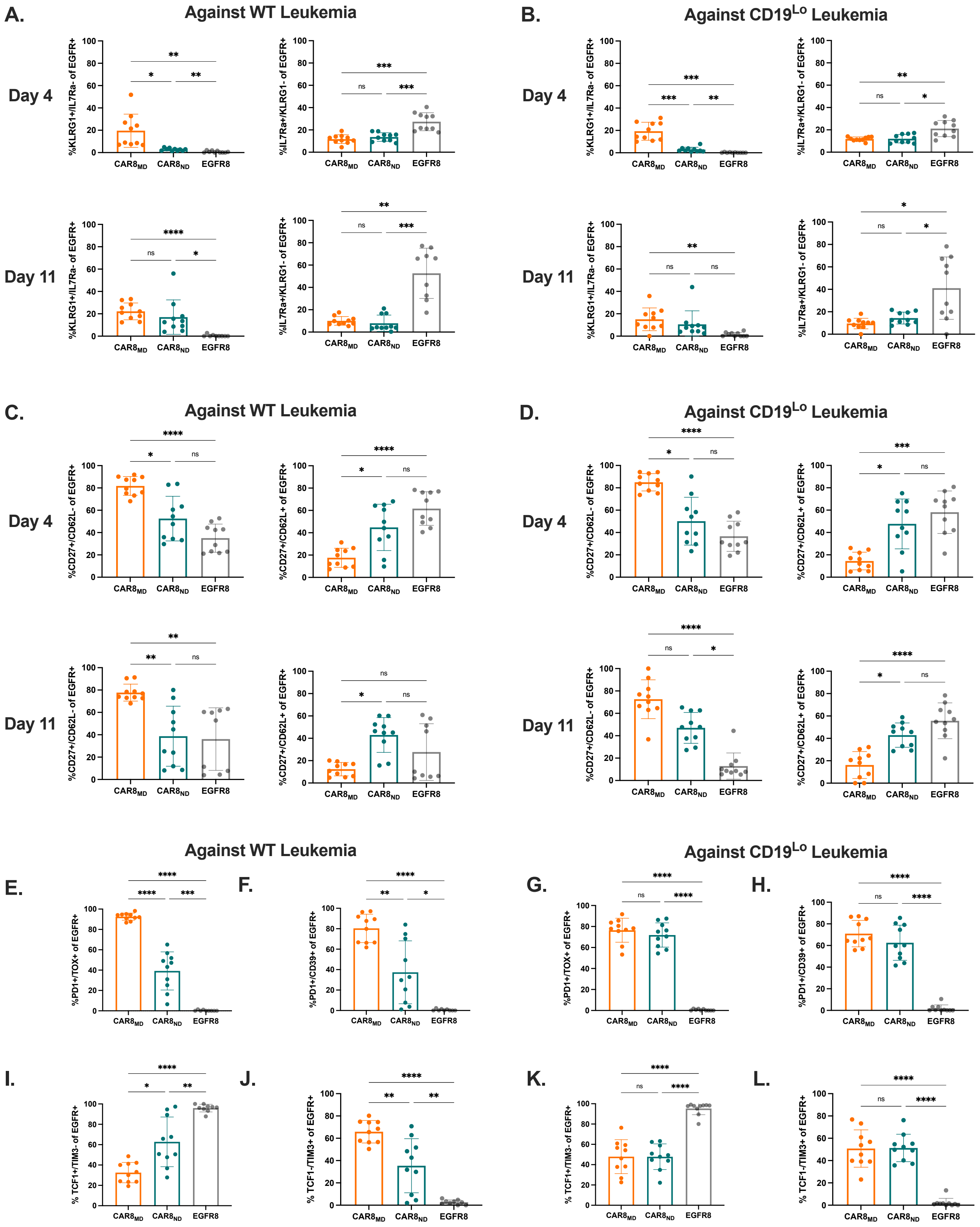

A.
Against WT Leukemia
B.
Against CD19Lo Leukemia
Day 4
Day 11
%KLRG1+/IL7Ra- of EGFR+
%IL7Ra+/KLRG1- of EGFR+
C.
Against WT Leukemia
D.
Against CD19Lo Leukemia
Day 4
Day 11
%CD27+/CD62L- of EGFR+
%CD27+/CD62L+ of EGFR+
Against WT Leukemia
Against CD19Lo Leukemia
E.
F.
G.
H.
%PD1+/TOX+ of EGFR+
%PD1+/CD39+ of EGFR+
I.
J.
K.
L.
% TCF1+/TIM3- of EGFR+
% TCF1-/TIM3+ of EGFR+
CAR8MD
CAR8ND
EGFR8

Figure S5: Additional *in vivo* effector/memory and exhaustion phenotyping at low CAR dose (Related to Figure 3).

All data in this figure are from experiments with the 3e5 EGFR+ cell dose. **S5A:** Proportions of CAR8 with the short-lived effector cell (SLEC, IL7Ra-/KLRG1+) or memory precursor effector cell (MPEC, IL7Ra+/KLRG1-) phenotypes at the indicated timepoint against WT leukemia. **S5B:** Proportions of CAR8 with the short-lived effector cell (SLEC, IL7Ra-/KLRG1+) or memory precursor effector cell (MPEC, IL7Ra+/KLRG1-) phenotypes at the indicated timepoint against $CD19^{Lo}$ leukemia. **S5C:** Proportions of CAR8 with the effector memory precursor (EMP, CD27+/CD62L-) or central memory precursor (CMP, CD27+/CD62L+) phenotypes at the indicated timepoint against WT leukemia. **S5D:** Proportions of CAR8 with the effector memory precursor (EMP, CD27+/CD62L-) or central memory precursor (CMP, CD27+/CD62L+) phenotypes at the indicated timepoint against $CD19^{Lo}$ leukemia. Figures S4E-L display proportions of CAR8 with the indicated phenotype at 11 days post-CAR injection against either WT (left, E,F,I,J) or $CD19^{Lo}$ (right, G,H,K,L) leukemia. **S5E & G:** PD1+/TOX+ **S5F & H:** PD1+/CD39+ **S5I & K:** TCF1+/TIM3- **S5J & L:** TCF1-/TIM3+. Data are from 2 pooled, independent experiments with n=10 mice per condition. Data represent mean +/- SD. * $p<0.05$, ** $p<0.01$, *** $p<0.001$, **** $p<0.0001$.

Figure S6

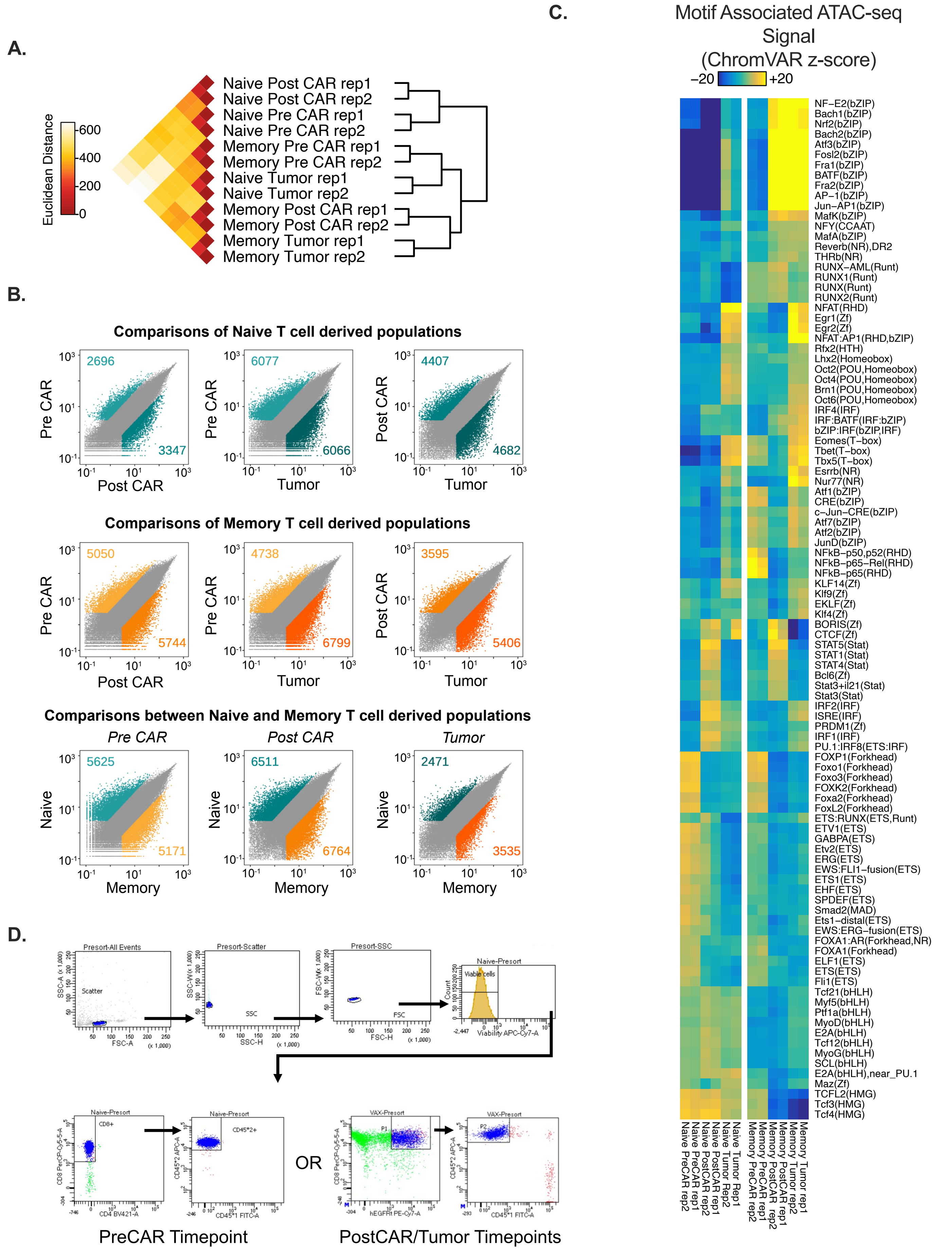
A.
Euclidean Distance
600
400
200
0
Naive Post CAR rep1
Naive Post CAR rep2
Naive Pre CAR rep1
Naive Pre CAR rep2
Memory Pre CAR rep1
Memory Pre CAR rep2
Naive Tumor rep1
Naive Tumor rep2
Memory Post CAR rep1
Memory Post CAR rep2
Memory Tumor rep1
Memory Tumor rep2
B.
Comparisons of Naive T cell derived populations
Pre CAR
Post CAR
2696
3347
6077
6066
Tumor
4407
4682
Comparisons of Memory T cell derived populations
5050
5744
4738
6799
3595
5406
Comparisons between Naive and Memory T cell derived populations
Pre CAR
Post CAR
Tumor
Naive
Memory
5625
5171
6511
6764
2471
3535
C.
Motif Associated ATAC-seq Signal (ChromVAR z-score)
−20
+20
NF−E2(bZIP)
Bach1(bZIP)
Nrf2(bZIP)
Bach2(bZIP)
Atf3(bZIP)
Fosl2(bZIP)
Fra1(bZIP)
BATF(bZIP)
Fra2(bZIP)
AP−1(bZIP)
Jun−AP1(bZIP)
MafK(bZIP)
NFY(CCAAT)
MafA(bZIP)
Reverb(NR),DR2
THRb(NR)
RUNX−AML(Runt)
RUNX1(Runt)
RUNX(Runt)
RUNX2(Runt)
NFAT(RHD)
Egr1(Zf)
Egr2(Zf)
NFAT:AP1(RHD,bZIP)
Rfx2(HTH)
Lhx2(Homeobox)
Oct2(POU,Homeobox)
Oct4(POU,Homeobox)
Brn1(POU,Homeobox)
Oct6(POU,Homeobox)
IRF4(IRF)
IRF:BATF(IRF:bZIP)
bZIP:IRF(bZIP,IRF)
Eomes(T−box)
Tbet(T−box)
Tbx5(T−box)
Esrrb(NR)
Nur77(NR)
Atf1(bZIP)
CRE(bZIP)
c−Jun−CRE(bZIP)
Atf7(bZIP)
Atf2(bZIP)
JunD(bZIP)
NFkB−p50,p52(RHD)
NFkB−p65−Rel(RHD)
NFkB−p65(RHD)
KLF14(Zf)
Klf9(Zf)
EKLF(Zf)
Klf4(Zf)
BORIS(Zf)
CTCF(Zf)
STAT5(Stat)
STAT1(Stat)
STAT4(Stat)
Bcl6(Zf)
Stat3+il21(Stat)
Stat3(Stat)
IRF2(IRF)
ISRE(IRF)
PRDM1(Zf)
IRF1(IRF)
PU.1:IRF8(ETS:IRF)
FOXP1(Forkhead)
Foxo1(Forkhead)
Foxo3(Forkhead)
FOXK2(Forkhead)
Foxa2(Forkhead)
FoxL2(Forkhead)
ETS:RUNX(ETS,Runt)
ETV1(ETS)
GABPA(ETS)
Etv2(ETS)
ERG(ETS)
EWS:FLI1−fusion(ETS)
ETS1(ETS)
EHF(ETS)
SPDEF(ETS)
Smad2(MAD)
Ets1−distal(ETS)
EWS:ERG−fusion(ETS)
FOXA1:AR(Forkhead,NR)
FOXA1(Forkhead)
ELF1(ETS)
ETS(ETS)
Fli1(ETS)
Tcf21(bHLH)
Myf5(bHLH)
Ptf1a(bHLH)
MyoD(bHLH)
E2A(bHLH)
Tcf12(bHLH)
MyoG(bHLH)
SCL(bHLH)
E2A(bHLH),near_PU.1
Maz(Zf)
TCFL2(HMG)
Tcf3(HMG)
Tcf4(HMG)
Naive PreCAR rep1
Naive PreCAR rep2
Naive PostCAR rep1
Naive PostCAR rep2
Naive Tumor Rep1
Naive Tumor Rep2
Memory PreCAR rep1
Memory PreCAR rep2
Memory PostCAR rep1
Memory PostCAR rep2
Memory Tumor rep1
Memory Tumor rep2
D.
Presort-All Events
Scatter
FSC-A
Presort-Scatter
SSC
SSC-H
Presort-SSC
FSC
FSC-H
Naive-Presort
Viable cells
Viability APC-Cy7-A
Count
CD8+
CD4 BV421-A
CD8 PerCP-Cy5-5-A
CD45*2+
CD45*1 FITC-A
CD45*2 APC-A
OR
VAX-Presort
P1
hEGFR PE-Cy7-A
P2
PreCAR Timepoint
PostCAR/Tumor Timepoints

Figure S6: Additional analyses of ATAC-seq data (Related to Figure 4).

All analyses in this figure are from the same timeline/experimental layout described in Figure 4A. **S6A:** Inter-replicate Euclidian distance of voom-normalized ATAC-seq counts per peak between biological replicates. **S6B:** Pairwise comparisons of differentially accessible chromatin regions within conditions between different timepoints of the same condition, or between different conditions at each timepoint. Data points are mean of voom-normalized ATAC-seq counts per peak between biological replicates of each group. **S6C:** Heatmap of motif-associated ChromVAR deviation z-scores patterns of motif-associated ATAC-seq signal for indicated transcription factors. List comprises all significant differentially accessible comparisons. **S6D:** Representative gating for sorting of cells in sequencing experiments.

Figure S7

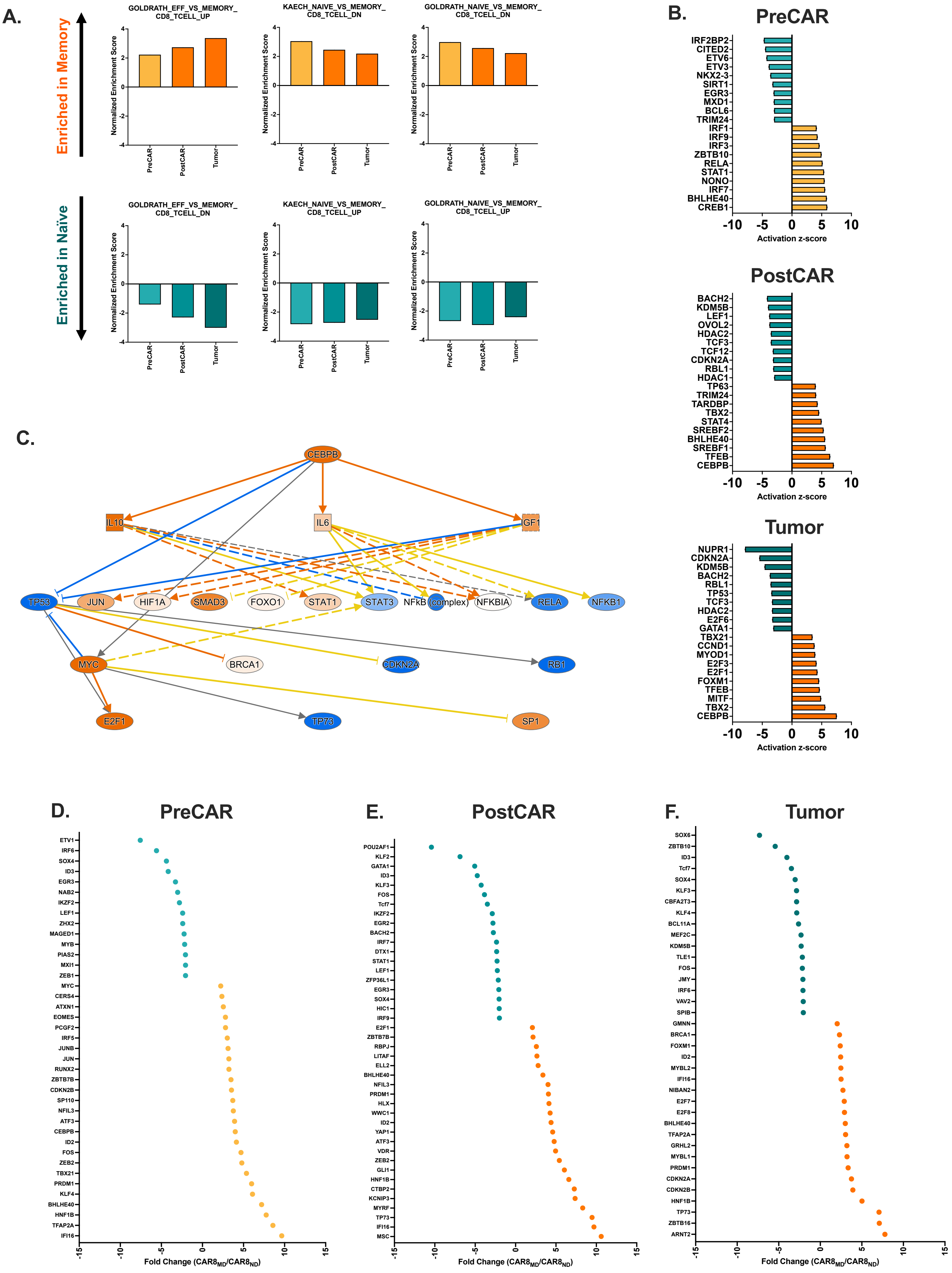

A.
Enriched in Memory
Enriched in Naïve
GOLDRATH_EFF_VS_MEMORY_CD8_TCELL_UP
KAECH_NAIVE_VS_MEMORY_CD8_TCELL_DN
GOLDRATH_NAIVE_VS_MEMORY_CD8_TCELL_DN
GOLDRATH_EFF_VS_MEMORY_CD8_TCELL_DN
KAECH_NAIVE_VS_MEMORY_CD8_TCELL_UP
GOLDRATH_NAIVE_VS_MEMORY_CD8_TCELL_UP
Normalized Enrichment Score
PreCAR
PostCAR
Tumor
B.
PreCAR
IRF2BP2
CITED2
ETV6
ETV3
NKX2-3
SIRT1
EGR3
MXD1
BCL6
TRIM24
IRF1
IRF9
IRF3
ZBTB10
RELA
STAT1
NONO
IRF7
BHLHE40
CREB1
Activation z-score
PostCAR
BACH2
KDM5B
LEF1
OVOL2
HDAC2
TCF3
TCF12
CDKN2A
RBL1
HDAC1
TP63
TRIM24
TARDBP
TBX2
STAT4
SREBF2
BHLHE40
SREBF1
TFEB
CEBPB
Activation z-score
Tumor
NUPR1
CDKN2A
KDM5B
BACH2
RBL1
TP53
TCF3
HDAC2
E2F6
GATA1
TBX21
CCND1
MYOD1
E2F3
E2F1
FOXM1
TFEB
MITF
TBX2
CEBPB
Activation z-score
C.
CEBPB
IL10
IL6
IGF1
TP53
JUN
HIF1A
SMAD3
FOXO1
STAT1
STAT3
NFkB (complex)
NFKBIA
RELA
NFKB1
MYC
BRCA1
CDKN2A
RB1
E2F1
TP73
SP1
D.
PreCAR
ETV1
IRF6
SOX4
ID3
EGR3
NAB2
IKZF2
LEF1
ZHX2
MAGED1
MYB
PIAS2
MXI1
ZEB1
MYC
CERS4
ATXN1
EOMES
PCGF2
IRF5
JUNB
JUN
RUNX2
ZBTB7B
CDKN2B
SP110
NFIL3
ATF3
CEBPB
ID2
FOS
ZEB2
TBX21
PRDM1
KLF4
BHLHE40
HNF1B
TFAP2A
IFI16
Fold Change (CAR8MD/CAR8ND)
E.
PostCAR
POU2AF1
KLF2
GATA1
ID3
KLF3
FOS
Tcf7
IKZF2
EGR2
BACH2
IRF7
DTX1
STAT1
LEF1
ZFP36L1
EGR3
SOX4
HIC1
IRF9
E2F1
ZBTB7B
RBPJ
LITAF
ELL2
BHLHE40
NFIL3
PRDM1
HLX
WWC1
ID2
YAP1
ATF3
VDR
ZEB2
GLI1
HNF1B
CTBP2
KCNIP3
MYRF
TP73
IFI16
MSC
Fold Change (CAR8MD/CAR8ND)
F.
Tumor
SOX6
ZBTB10
ID3
Tcf7
SOX4
KLF3
CBFA2T3
KLF4
BCL11A
MEF2C
KDM5B
TLE1
FOS
JMY
IRF6
VAV2
SPIB
GMNN
BRCA1
FOXM1
ID2
MYBL2
IFI16
NIBAN2
E2F7
E2F8
BHLHE40
TFAP2A
GRHL2
MYBL1
PRDM1
CDKN2A
CDKN2B
HNF1B
TP73
ZBTB16
ARNT2
Fold Change (CAR8MD/CAR8ND)

Figure S7: Additional analyses of RNA-seq data (Related to Figure 5).

All analyses in this figure are from the same timeline/experimental layout described in Figure 4A. **S7A:** Normalized enrichment scores from GSEA of differentially enriched genesets between indicated CD8+ T cell subsets after LCMV-armstrong acute viral infection[24, 25]. **S7B:** Top transcriptional activators predicted to be activated and driving differential transcriptional state between naïve versus memory-derived cells at the indicated timepoint, as predicted by Qiagen Ingenuity Pathway Analysis[28] (IPA). **S7C:** IPA activation map for the Cebpb transcription factor, the top predicted driver of transcriptional state in memory-derived cells at the PostCAR and Tumor timepoints. **S7D-F:** Top differentially expressed transcription factors, at the indicated timepoint. All statistics performed using DESeq2 with filtering threshold at 10, log2foldchange >2 and padj > 0.05.

Figure S8

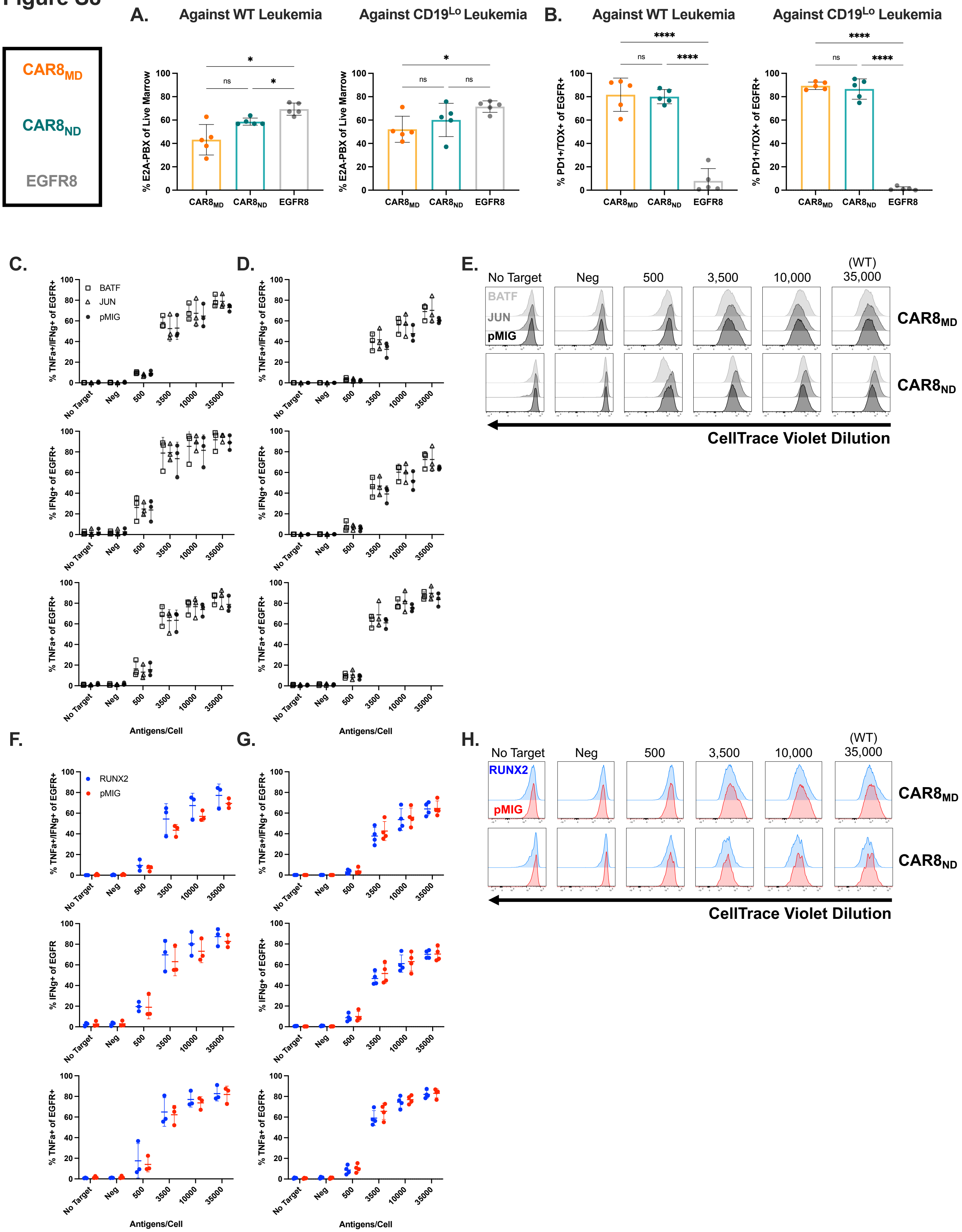
A.
Against WT Leukemia
Against CD19Lo Leukemia
B.
Against WT Leukemia
Against CD19Lo Leukemia
CAR8MD
CAR8ND
EGFR8
% E2A-PBX of Live Marrow
% PD1+/TOX+ of EGFR+
ns
*
****
C.
D.
E.
BATF
JUN
pMIG
% TNFa+/IFNg+ of EGFR+
% IFNg+ of EGFR+
% TNFa+ of EGFR+
No Target
Neg
500
3500
10000
35000
Antigens/Cell
No Target
Neg
500
3,500
10,000
(WT)
35,000
CellTrace Violet Dilution
F.
G.
H.
RUNX2
pMIG
% IFNg+ of EGFR

Figure S8 (Related to Figure 6): Characterization of 1e5 CAR T cell dose *in vivo* experiments and *in vitro* comparisons of BATF, JUN or RUNX2 overexpressing cells to pMIG.

All analyses in this figure are the same timeline/experimental layout described in Figure 3A except with 1e5 CAR+ cell dose, at 11 days post-CAR timepoint. S8A-B are characterization of the 1e5 cell dose with standard T cell groups (no ectopic transcription factor expression). **S8A:** Leukemia burden. **S8B:** Proportions of CAR8 with the PD1+/TOX+ phenotype. Data in S7A-B are from 1 experiment with n=5 mice per condition. * $p<0.05$, ** $p<0.01$, *** $p<0.001$, **** $p<0.0001$. **S8C-D:** Quantification of intracellular cytokine staining of IFNg and TNFa after 6 hour co-culture assay, % positive of EGFR+, for memory (C) or naïve-derived (D) cells cotransduced with BATF, JUN or pMIG. Data in S8C-D are from 3 independent experiments. **S8E:** Proliferation as measured by dilution of CellTrace Violet dye dilution of EGFR+ cells after 72 hour co-culture assay, for memory or naïve derived cells cotransduced with BATF, JUN or pMIG. Data representative of 3 independent experiments. **S8F-G:** Quantification of intracellular cytokine staining of IFNg and TNFa after 6 hour co-culture assay, % positive of EGFR+, for memory (C) or naïve-derived (D) cells cotransduced with RUNX2 or pMIG. Data in S8C-D are from 3-4 independent experiments. **S8H:** Proliferation as measured by dilution of CellTrace Violet dye dilution of EGFR+ cells after 72 hour co-culture assay, for memory or naïve derived cells cotransduced with RUNX2 or pMIG. Data representative of 3 independent experiments. No statistically significant differences were found between BATF, JUN or RUNX2 engineered CAR T cells and pMIG control T cells for in vitro data. Data represent mean +/- SD.

Figure S9

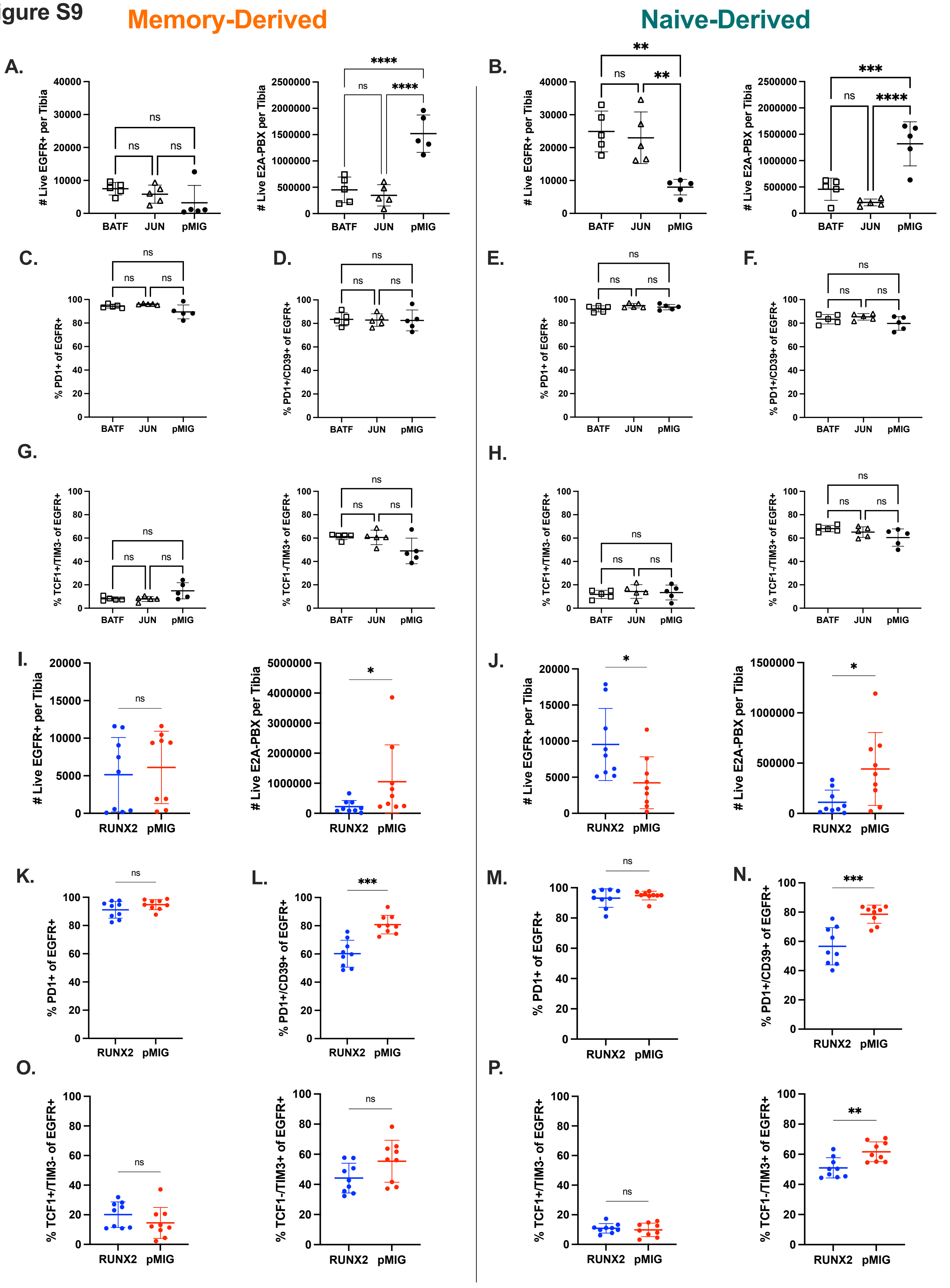
Memory-Derived
Naive-Derived
A.
B.
C.
D.
E.
F.
G.
H.
I.
J.
K.
L.
M.
N.
O.
P.
Live EGFR+ per Tibia
Live E2A-PBX per Tibia
% PD1+ of EGFR+
% PD1+/CD39+ of EGFR+
% TCF1+/TIM3- of EGFR+
% TCF1-/TIM3+ of EGFR+
BATF
JUN
pMIG
RUNX2
ns
*
**
***
****

Figure S9 (Related to Figure 6): Counts and additional exhaustion phenotyping data for BATF, JUN or RUNX2 overexpression *in vivo* experiments.

All analyses in this figure are done on the same experiments described in Figure 6. Counts data was generated by flushing a single tibia and using total tibia counts and cytometer proportions data to calculate CAR and leukemia cell counts per tibia. **S9A-B:** CAR and leukemia counts for BATF or JUN overexpressing memory (A) or naïve-derived (B) CAR T cells compared to pMIG control. **S9C-H:** Proportions of EGFR+ cells from BATF, JUN or pMIG CAR8 with the indicated phenotype. S9C,D,G are memory-derived cells, S9E,F,H are naïve-derived cells. **S9C,E:** PD1+ **S9D,F:** PD1+/CD39+ **S9G-H:** Indicated TCF1/TIM3 phenotype. Data in S9A-H are from one experiment with n=5 mice per condition. **S9I-J:** CAR and leukemia counts for RUNX2 overexpressing memory (A) or naïve-derived (B) CAR T cells compared to pMIG control. **S9K-N:** Proportions of EGFR+ cells from RUNX2 or pMIG CAR8 with the indicated phenotype. S9C,D,G are memory-derived cells, S9E,F,H are naïve-derived cells. **S9K,M:** PD1+ **S9L,N:** PD1+/CD39+ **S9O,P:** Indicated TCF1/TIM3 phenotype. Data in S9I-P are from 2 pooled, independent experiments with n=9 mice per condition. Data represent mean +/- SD. * $p<0.05$, ** $p<0.01$, *** $p<0.001$, **** $p<0.0001$.
